# Dual roles of genes required for intrinsic resistance to clarithromycin in evasion of killing by serum complement in *Haemophilus influenzae*

**DOI:** 10.1128/iai.00369-26

**Published:** 2026-08-06

**Authors:** Cameron Huhn, Justine Dees, Jeffrey Gawronski, Asma Ul Taslim, Sandy Wong, Brian Akerley

**Affiliations:** 1 Department of Cell and Molecular Biology, University of Mississippi Medical Center 189400, Jackson, Mississippi, USA; 2 Department of Microbiology and Physiological Systems, University of Massachusetts Medical School 200789, Worcester, Massachusetts, USA; University of California Davis, Davis, California, USA

**Keywords:** *Haemophilus influenzae*, NTHi, clarithromycin, *omp26*, *orfH*, lung infection, mouse model, HITS, Tn-seq, serum resistance

## Abstract

Macrolide antibiotics are commonly prescribed to treat *Haemophilus influenzae* respiratory tract infections. Studies have primarily focused on emerging *H. influenzae* strains with acquired macrolide resistance, while the bacterium’s intrinsic resistance to antibiotics has been underexamined. Here, we used a genome-wide approach of transposon insertion-site sequencing to screen an *H. influenzae* mutant library grown in sub-inhibitory doses of the macrolide antibiotic clarithromycin (CLR) to identify 33 genes involved in intrinsic CLR resistance. Almost half of these genes are also needed for survival in the mouse lung. We focused on candidate genes necessary for both intrinsic macrolide resistance and lung survival. Two of these genes affect the outer-membrane composition of *H. influenzae*, *orfH* and *omp26*. Deletions of these genes in Rd and nontypeable *H. influenzae* clinical isolates, Hi375 and NT127, conferred sensitivity to CLR and polymyxin B and increased membrane permeability to ethidium bromide (EtBr). The *omp26* mutant was sensitive to killing by human serum. Deletions of *orfH* or *omp26* in an *acrR* mutant strain overexpressing a multidrug efflux pump abrogated resistance of the *acrR* mutant to CLR and restored permeability to EtBr. Thus, deletion of these genes not only mitigates the effects of an acquired resistance mechanism but also remarkably overrides it. Complementation of these deletion mutations restored CLR resistance and decreased permeability to EtBr. Our results indicate that the subset of genes with dual roles in intrinsic resistance and host lung survival may provide potential novel combination antimicrobial therapeutic targets.

## INTRODUCTION

The gram-negative bacterium *Haemophilus influenzae* colonizes the nasopharynx of 20%–80% of healthy children by the age of 5 and 20%–30% of healthy adults (1, 2), providing a reservoir for pathogenic infections. *H. influenzae* isolates are classified as typeable encapsulated strains, of which type b (Hib) is the main pathogenic serotype, and unencapsulated nontypeable *H. influenzae* (NTHi). NTHi is a common cause of upper and lower respiratory infections in humans, including pneumonia, bronchitis, cystic fibrosis lung infections, exacerbations of chronic obstructive pulmonary disease (COPD), sinusitis, and otitis media. While a Hib vaccine containing the type b capsular polysaccharide has been highly effective in geographical locations where it is used, it has no effect on NTHi, and there is no effective vaccine available against NTHi, which continues to cause a significant burden of disease. Otitis media is the leading cause for pediatric antibiotic prescriptions in the United States (3). Moreover, COPD is the third leading cause of death worldwide, and in the United States, it is the sixth leading cause of death and the most common chronic lung disease (4–6). NTHi is the most common exacerbator of COPD, responsible for ~50% of bacterial exacerbations and ~30% of all exacerbations (7–9). NTHi takes advantage of the altered lung environment provided by COPD, leading to bacteria-induced exacerbations and persistence during stable periods, both accelerating the rate of deterioration of the lungs (10, 11).

Clinicians often treat respiratory infections with the β-lactam antibiotic amoxicillin; however, a common problem when treating *H. influenzae* infections, especially COPD exacerbations and otitis media, is the failure of antibiotic efficacy (12, 13). Approximately 30% of clinically isolated *H. influenzae* strains are resistant to β-lactams (14, 15), partly due to β-lactamase production (16, 17); moreover, β-lactamase-negative resistant strains are becoming more common (14, 18–21). Macrolide antibiotics, such as clarithromycin (CLR) or azithromycin, are also used to treat respiratory infections and are among the first-line treatment options for acute exacerbated COPD (22). However, an upward estimate of almost 40% of clinical isolates are resistant to CLR (23–26), and strains from infections have been shown to develop resistance during the course of treatment with the macrolide azithromycin (27). The mechanism responsible for macrolide antimicrobial activity is generally considered to be their ability to target the 50S subunit of the ribosome to stop translation (28). In addition to their antimicrobial activity, macrolides have an immunomodulatory effect on the host as they can decrease the levels of inflammatory cytokines and immune cells in various *in vivo* models (29–32).

Macrolide resistance in *H. influenzae* involves acquired mechanisms and intrinsic factors. Deregulation of the multidrug efflux pump system *acrAB* via nonsense mutations acquired in the negative regulator *acrR* was found in ~20% of resistant isolates (33, 34), and an estimated 85% of NTHi resistant isolates had substitutions in ribosomal proteins L4 or L22, the ribozyme 23S rRNA, or in the multidrug efflux pump component AcrB (34). Mutations to the ribosomal proteins or 23S rRNA modify the macrolide target within the peptidyl transferase center and prevent protein synthesis inhibition (35–38). Substitutions in AcrB may contribute to resistance by enhancing substrate recognition and increasing efflux (33, 34). Intrinsic mechanisms have been identified as well, including macrolide resistance through normal expression of *acrAB* (39), but no published studies have assessed *H. influenzae’s* intrinsic resistance on a genome-wide scale. Therefore, we set out to answer the following questions: which *H. influenzae* genes are intrinsic determinants of clarithromycin resistance, and can these genes be used as targets to mitigate acquired resistance?

The broad-based approach to understanding bacterial fitness, transposon insertion site sequencing, with variations termed HITS (40), INseq (41), Tn-seq (42), or TraDIS (43), allows for the identification of genes—on the genome-wide level—required for fitness in an experimental condition or between different conditions of interest. This method simultaneously compares the fitness of each mutant within a pool of thousands of transposon mutants. Before and after the growth of the pool of mutants in a defined condition, the relative abundance of each mutant is determined by sequencing the DNA adjacent to the transposon insertion, and mutants that become less abundant are identified as having reduced fitness in that condition. We employed this approach to determine the genes involved in intrinsic resistance of *H. influenzae* to two different doses of CLR. We identified 33 genes involved in resistance to CLR and found that most of these genes are conserved and present in the core genome of *H. influenzae*. We focused our attention on genes encoding potential drug targets necessary for both intrinsic macrolide resistance and survival in the mouse lung. Genes that are required for both CLR resistance and bacterial survival during infection are of particular interest, as targeting their products with inhibitory compounds could improve both the direct therapeutic efficacy of CLR and render *H. influenzae* less capable of infection.

The roles of two of these genes involved in outer-membrane composition were evaluated in three wild-type strain backgrounds and in the presence of a mutation in *acrR* that commonly causes increased drug efflux in antibiotic-resistant clinical isolates. To investigate functions of these genes in infection that might be targeted to provide synergistic effects with antibiotics *in vivo*, we first evaluated their roles in resistance to serum complement, as complement evasion is an important defense mechanism of *H. influenzae*. To begin to extend these results to additional conditions that bacteria are expected to encounter during clinical therapy, we used defined mutants to evaluate potential antagonistic or synergistic effects caused by exposure to sub-inhibitory (sub-minimum inhibitory concentration [sub-MIC]) concentrations of CLR on subsequent CLR and antimicrobial peptide resistance, using polymyxin B as a model for the latter. These *in vitro* correlates of immune evasion could be used to design screens for inhibitory compounds acting on the identified proteins. Overall, these data provide potential novel combination therapeutic targets.

## RESULTS

### Identification of CLR intrinsic resistance genes in *H. influenzae*

**Fig 1:**
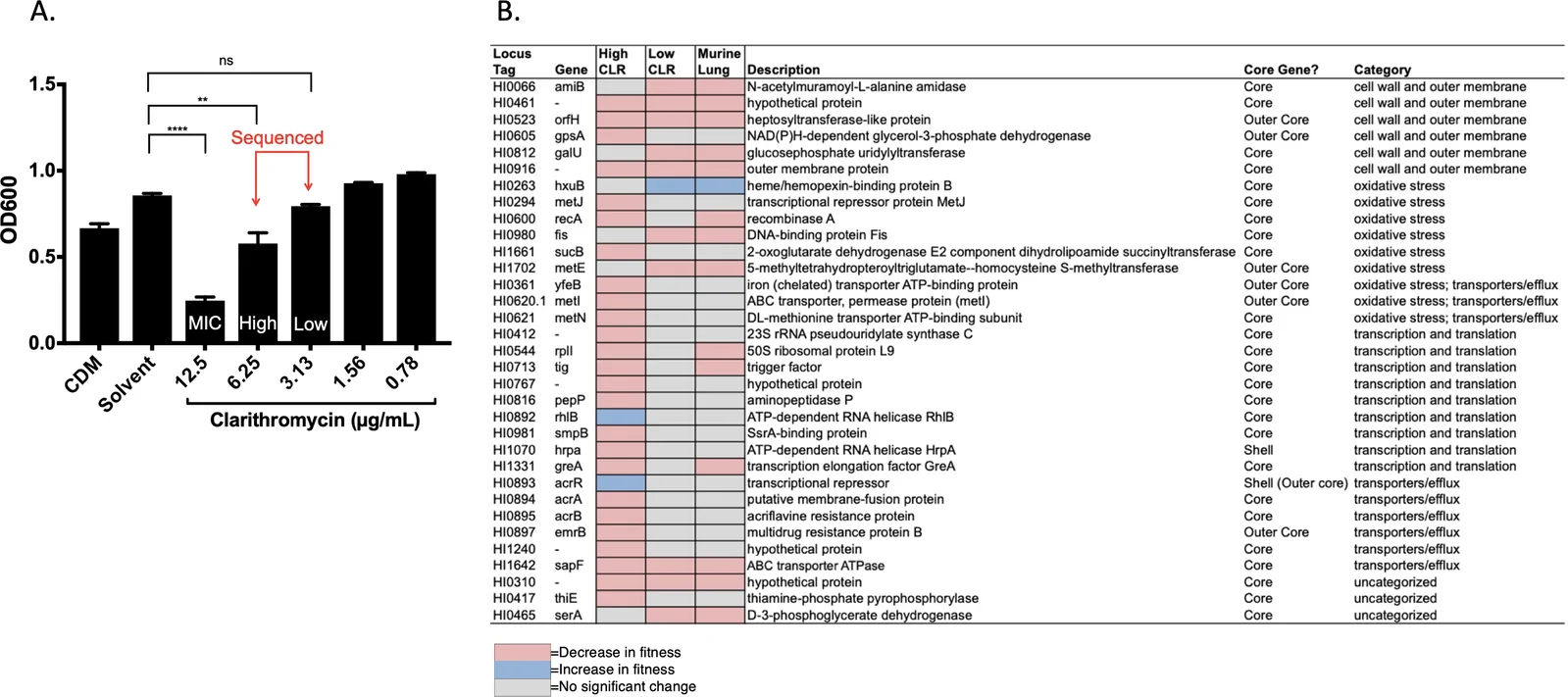
Thirty-three genes were identified that influenced clarithromycin resistance. (**A**) The bar graph shows the final growth yields of the *H. influenzae* Rd mutant library. The concentrations of clarithromycin selected for conditional comparisons of mutant libraries were those that affected growth significantly, 1/2 MIC (high), and those that did not affect growth significantly, 1/4 MIC (low), which are indicated by the red arrows. The bars represent the optical densities at 600 nm. The error bars represent the standard error of the mean. Differences were evaluated via ANOVA with Tukey’s multiple comparisons test (****P < 0.00005; **P < 0.005; and ns, not statistically significant). (**B**) Thirty-three genes influencing resistance to clarithromycin were cross-referenced to fitness determinants in the murine lung infection and pan-genome analysis (Tables S2 and S3). The locus tag for *H. influenzae* RdAW, gene name, and protein descriptions are included. The column labeled “Core Gene?” represents the results from the pan-genome analysis (Table S3).

To determine the genes necessary for intrinsic resistance to CLR in *Haemophilus influenzae*, we utilized the genome-wide transposon mutagenesis technique high-throughput insertion tracking by deep sequencing (HITS, also called Tn-Seq) to analyze over 70,000 mutants in the genetically tractable strain Rd background at two different concentrations of sub-inhibitory concentrations (sub-MIC) of CLR (Fig. 1A). We used sub-MIC concentrations because our aim was to discover susceptible mutants through negative selection. This approach contrasts with positive selection in CLR concentrations above the MIC, which would yield acquired resistance mutations. We grew our mutant library in a chemically defined medium (44) to facilitate follow-up metabolic studies by providing control over available metabolites, with various concentrations of CLR. We sequenced and identified fitness determinants in 1/2 MIC (high CLR) and 1/4 MIC (low CLR) concentrations of CLR relative to media alone (Fig. 1A). Genomic DNA (gDNA) was prepared from mutant libraries after selection and processed by a modified version of our HITS technique (Materials and Methods), and genes were analyzed for their roles in fitness in chemically defined medium alone or with CLR selection. First, genes were categorized as essential, growth-defective, non-essential, or growth-advantaged without CLR based on the resulting transposon mutant phenotype. Genes that were considered essential or growth-defective in the input library, or in the absence of treatment, were eliminated from our analysis of genes required for fitness in our chosen conditions (Table S1). We compared mutants in four conditions (chemically defined medium [CDM], CDM with solvent, CDM with 1/2 MIC CLR, and CDM with 1/4 MIC CLR) to the abundance of mutants in the input library, which was grown in rich media (sBHI) (Table S2). Genes were considered required for CLR resistance if the mutants displayed altered fitness in CDM with CLR but not in CDM alone or CDM with solvent. Based on this criterion, 33 genes were detected in the screen as influencing resistance to CLR (Fig. 1B; Table S3). In total, 20 genes (red shade) were required at 1/2 MIC of CLR (High), 5 at 1/4 MIC (Low), and 5 at both doses of CLR (Fig. 1B). In contrast, three genes (blue shade) appear to be detrimental for CLR resistance, as mutations in these genes enhanced fitness. Overall, genes were categorized into four broad functional categories: (i) cell wall and outer membrane, (ii) oxidative stress, (iii) transporters/efflux, and (iv) transcription and translation.

Interestingly, of the 33 genes identified, loss of 3 genes, HI0263 (*hxuB*), HI0892 (*rhlB*), and HI0893 (*acrR*), enhanced CLR resistance. Mutations in *hxuB* increased resistance at 1/4 MIC concentration of CLR. Categorized in the oxidative stress category, *hxuB*, which is involved in the secretion of HxuA, is part of the *oxyR* regulon and the heme utilization system (45, 46). In the presence of hydrogen peroxide, a source of oxidative stress, *hxuB* is upregulated (45), which indicates that *H. influenzae* might be experiencing oxidative stress in the presence of CLR. Categorized as being involved in transcription and translation, mutations in *rhlB*, whose gene product helps unwind dsRNA and degrade mRNA in *Escherichia coli* as part of the degradosome (47–49), increased resistance to CLR at 1/2 MIC concentration. In *E. coli*, deletion of *rhlB* resulted in increased mRNA half-life (50), which might allow for abnormally higher levels of expression for proteins that allow *H. influenzae* to escape the pressure of CLR-inhibited protein synthesis. Finally, it is noteworthy that mutations in *acrR*, the negative regulator of the efflux system *acrAB*, also increased resistance to CLR at 1/2 MIC concentrations. The efflux pump system AcrAB is known to be involved in intrinsic and acquired macrolide resistance (39). In clinical isolates, CLR resistance was attributed to frameshift mutations that introduced a premature stop codon in *acrR*, resulting in truncation of the *acrR* gene product and subsequent overexpression of *acrAB* (33).

Only five genes were necessary for fitness in both concentrations of CLR. Three of these mediate cell wall and outer membrane biogenesis: HI0523 (*orfH*), which encodes a heptosyltransferase responsible for the third heptose extension of the inner-core lipooligosaccharide (LOS) (51); HI0461, implicated in LOS modification (52); and HI0916 (*omp26*), which encodes a 26-kDa protein that has been detected on the outer membrane (53). The remaining two are HI1642 (*sapF*), classified under the transporters/efflux category and encoding an ABC transporter ATPase implicated in heme uptake and biofilm formation (54), and HI0310, a DUF5339 domain-containing protein that has an unknown function and was uncategorized. Interestingly, *orfH* and HI0461 are also required for infection via complement resistance in *H. influenzae* (40, 52, 55). The gene product of *omp26* is a vaccine candidate that has been shown to be protective against heterologous strains and enhanced IgG and IgA production in rats (53). Loss of *sapF* attenuated persistence in the chinchilla nasopharynx and middle ear (54). These results suggested an intriguing level of overlap between the set of genes required for resistance to CLR and those involved in infection.

### Requirement for resistance genes in murine lung infection

To investigate the requirement for genes necessary for resistance to CLR during lung infections, we comprehensively determined genes necessary for fitness in the murine lung model. Genes necessary for both CLR and lung infections would provide stronger candidates as drug targets to potentially work synergistically with CLR. Therefore, we infected murine lungs with the mutant library and determined which genes were required for fitness in comparison to the input mutant library grown in rich media (sBHI) using the HITS technique. We have previously performed HITS in the mouse lung during the development of this technique (40); however, Illumina technology has improved in quality and throughput since our initial study. Here, we report a 14-fold greater sequencing density, and 17 more genes are implicated in the lung infection model (Table S2). Out of the 33 genes identified as influencing CLR resistance, 14 were required for the murine lung (Fig. 1B). All genes necessary or detrimental for survival in 1/4 MIC (low) concentrations of CLR were also needed in the mouse lung model, indicating that some of the selective pressures in the lung may be similar to those generated by lower CLR concentrations. Of the 20 genes required for survival in 1/2 MIC (high) concentrations of CLR, 4 were also required in the murine lung model, with 5 required for both concentrations of CLR and the murine lung model.

### Pan-genome analysis

Drug targets with high conservation across strains are highly attractive, but *H. influenzae* is a diverse bacterial species, with at most ~50% conservation across strains from one estimate (56), and based on other studies, possibly even lower conservation (38%–42%) (57, 58). Thus, we sought to determine whether these CLR resistance genes are conserved among *H. influenzae* strains. Although previous studies have identified the *H. influenzae* core genome (56–58), the full pan-genome data set is not publicly available; therefore, we constructed a pan-genome for this study. We built a pan-genome with 112 high-quality *H. influenzae* genomes (Table S4) and determined whether these genes are in the core genome using the pan-genome analysis pipeline, Roary (59). Because Roary was designed to build comparisons sequentially in an arbitrary order, each run can potentially generate a slightly different pan-genome. Therefore, we ran the analysis five times (Tables S5 and S6) and selected the mode to represent the number of genomes containing each gene. We used a stringent cutoff of 90% for the blastp percentage identity. Our pan-genome analysis identified some likely sequencing errors in Rd KW20 and other *H. influenzae* genomes, which are discussed in Text S1. Roary assigned genes to four categories based on the percentage of genomes they were found in: core (99%–100%), outer core (95%–99%), shell (15%–95%), and cloud (0%–15%) (Table S3; Fig. 1B). Of the 33 genes influencing resistance to CLR, 25 were categorized as part of the core pan-genome (76%); 7 were part of the outer core (18%); and 1 was part of the shell (3%). None of the genes implicated in CLR resistance fell into the cloud category (Fig. 1B). Core genes accounted for 72.7% (16/22) of 1/2 MIC resistance genes, 83.3% (5/6) of 1/4 MIC resistance genes, and 80.0% (4/5) of resistance genes identified at both CLR concentrations (Fig. 1B). Therefore, most of the genes of *H. influenzae* contributing to CLR resistance are conserved.

### Validation of CLR resistance determinants

**Fig 2:**
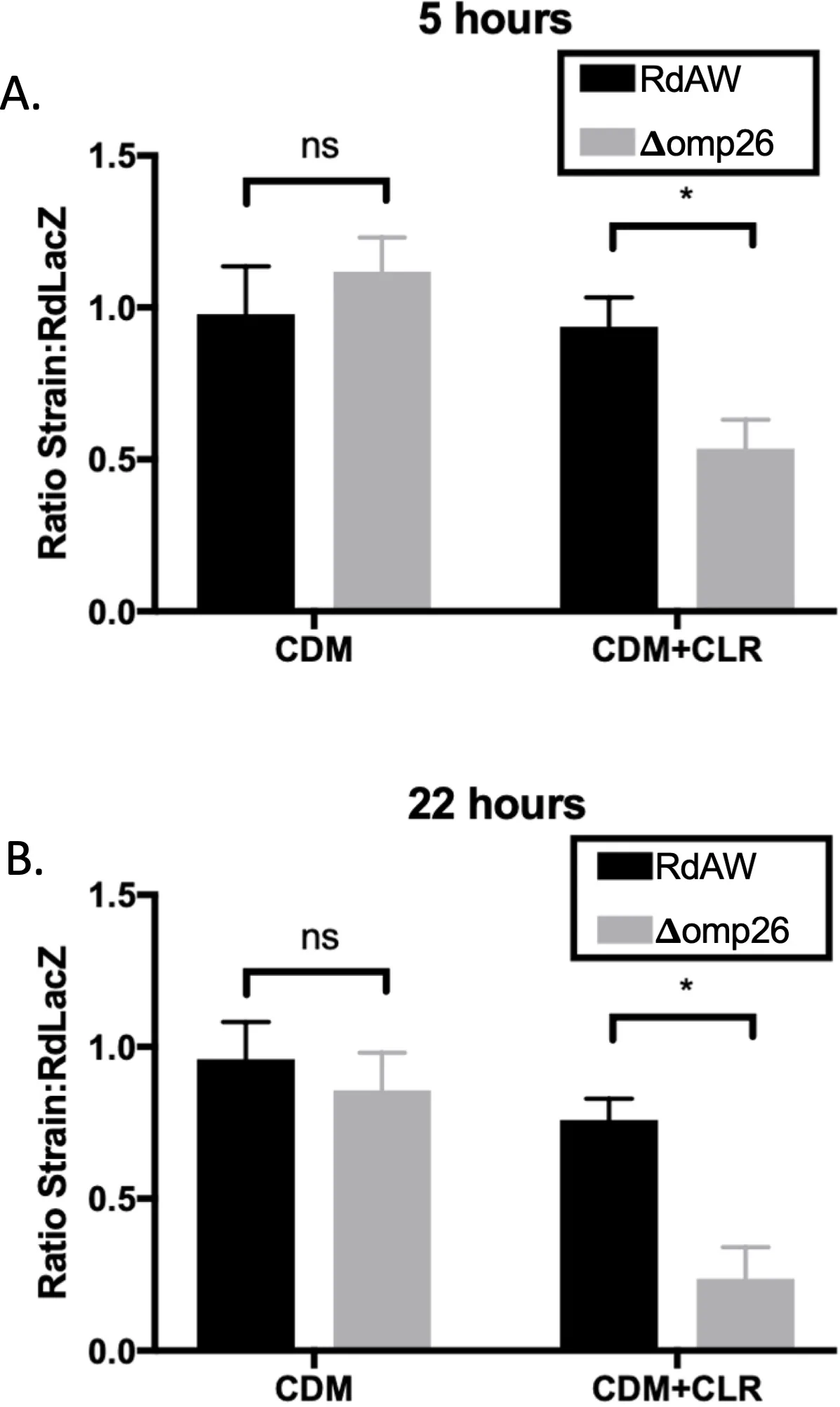
Omp26 was required for clarithromycin resistance in a competitive environment. An *H. influenzae* strain (RdlacZ) expressing *lacZ* ectopically in the *xyl* locus was used to compete with RdAW and Rd∆omp26. The strains were grown in either CDM or CDM + sub-MIC CLR (CDM + CLR) for (**A**) 5 h and (**B**) 22 h, then plated for colony counts on LacZ indicator agar (Materials and Methods). Black bars represent wild-type RdAW competitions, and gray bars represent RdΔomp26 competitions. Data shown are the ratios between the RdLacZ strain and RdAW or RdΔomp26. Differences were evaluated via ANOVA with Šidák’s multiple comparisons test (*, *P* < 0.05; ns, not statistically significant).

For validation of our HITS results for genes involved in CLR resistance, we selected genes based on the following criteria: the genes must be (i) required for fitness during growth in both concentrations of CLR, (ii) required for fitness during murine lung infection, and (iii) present in at least 95% of *H. influenzae* genomes. Five genes fit these criteria, and we selected three of them, all of which were involved or implicated in outer membrane composition: *orfH*, *omp26*, and HI0461. We created in-frame deletions of these genes by replacing them with antibiotic resistance cassettes in two different strains: RdAW and the nontypeable clinical isolate Hi375. We utilized minimum inhibitory concentration assays to validate their roles in CLR resistance. All mutants except RdΔomp26 were at least twofold more sensitive than their wild-type parents (Table S7). These results confirm the contribution of *orfH* and HI0461 to CLR resistance. Because mutants were tested in a transposon library, we hypothesized that RdΔomp26 would be more sensitive in a competition environment. To address this hypothesis, we performed competition assays in which we grew the wild-type RdAW or RdΔomp26 with the indicator strain RdlacZ, which is RdAW expressing *lacZ* ectopically in the *xyl* locus. We grew the co-cultures with and without sub-MIC concentrations of CLR and plated for colony-forming unit (CFU) determination at 5 and 22 h of growth. There was an insignificant difference between RdAW and RdlacZ after both 5 and 22 h of growth. In contrast, at 5 h, we saw a significant difference between RdΔomp26 and RdlacZ and a larger difference after 22 h (Fig. 2), confirming the necessity of *omp26* for growth in the presence of CLR in a competitive environment.

### Effect of deletions of CLR-resistant genes in various strains

Currently, while the traditional MIC microbroth assays are a useful method for rapidly screening bacteria for high levels of resistance to a wide variety of antibiotics, it does not allow for the resolution of detection for strains that have more moderate differences of resistance or sensitivity compared to reference strains, nor does it provide precision when obtaining the MIC due to the wide range of concentrations of antibiotic used in the assay. To obtain more exact values, we utilized a method, as described by Lambert and Pearson (60), with slight modifications, in which the MIC could be calculated from a curve fitting procedure (60). We used 0.8-fold dilutions of CLR instead of the traditional twofold dilutions to generate a tighter range of concentrations and grew the cells overnight. The final OD_600_ was plotted against the concentration of CLR, and the curve was fitted based on the modified Gompertz function, from which the MIC could be extrapolated (Materials and Methods).

**Fig 3:**
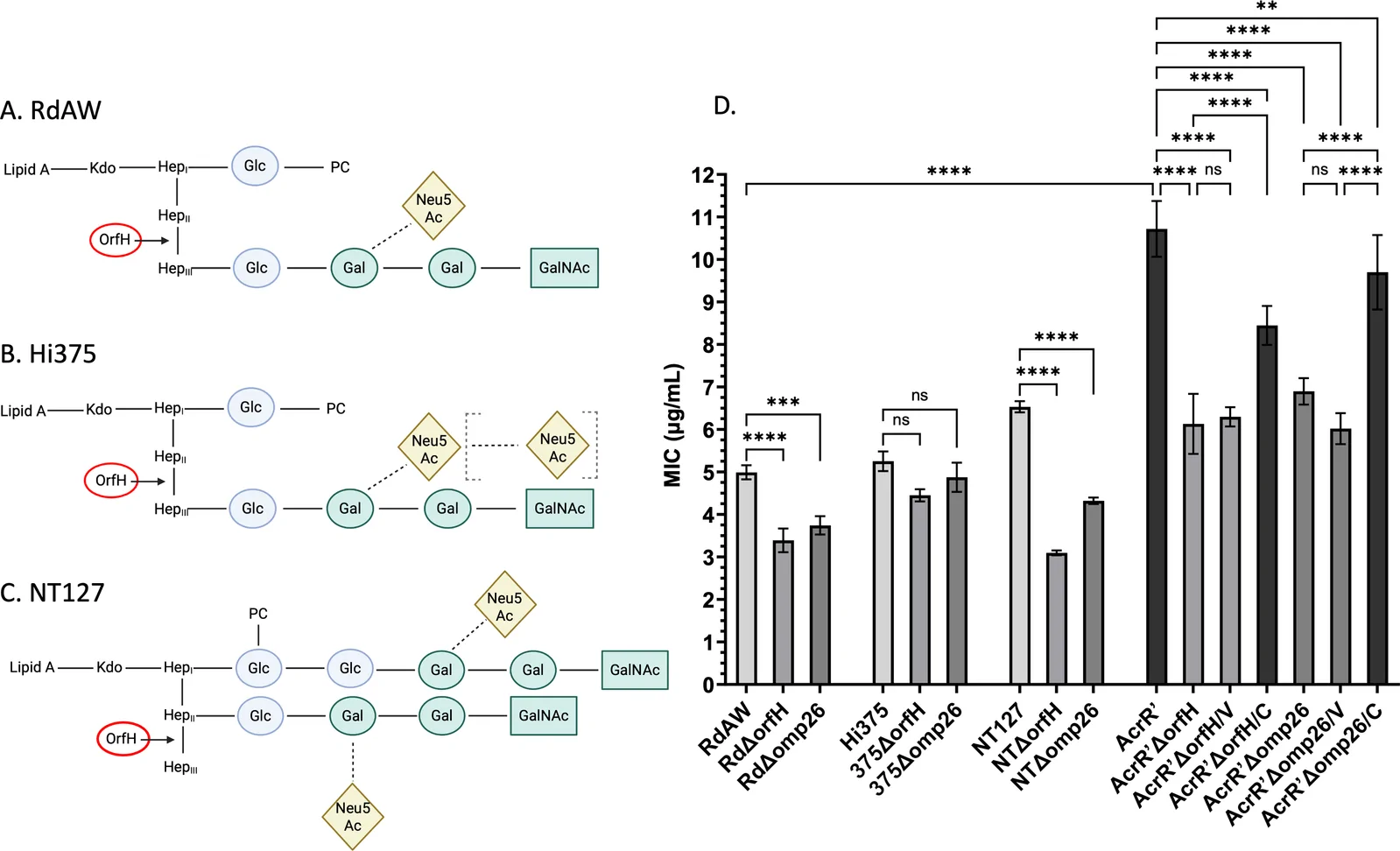
Intrinsic resistance genes are essential for wild-type levels of resistance and for the acquired increased resistance in AcrR′ strains. Each strain was grown overnight in sMH in the presence of clarithromycin. The endpoint OD_600_ was plotted against the concentration of clarithromycin, and the MIC was extrapolated from the curve of the line. Diagrams of the LOS structures of (**A**) RdAW (51, 64), (**B**) Hi375 (65), and (**C**) NT127 (66). Kdo, 2-keto-3-deoxyoctulosonic acid; Hep, heptose; Glc, glucose; Gal, galactose; GalNAc, N-acetylgalactosamine; Neu5Ac, sialic acid; and PC, phosphorylcholine. OrfH, which is responsible for the third heptose extension, is circled in red. (**D**) Results are shown as the average of the calculated MIC values, with the bars representing the standard error of the mean. Differences were evaluated via one-way ANOVA with Tukey’s multiple comparisons test (****, *P* < 0.0001; ***, *P* < 0.001; **, *P* < 0.01; and ns, not statistically significant).

We evaluated the MICs for RdAW, two nontypeable clinical isolates, Hi375 and NT127, and their isogenic *orfH* and *omp26* deletion mutants (Fig. 3 and Table 1). The MIC for Rd was 4.992 ± 0.1665 µg/mL, which is similar to the reported MIC for Rd when tested in Haemophilus Test Medium (8 μg/mL) (61–63). In Rd, a 32.1% decrease was seen when *orfH* was deleted and a 25% decrease when *omp26* was deleted, revealing only a moderate effect on sensitivity to CLR (Fig. 3D, far left). The MIC for Hi375 was calculated to be 5.252 ± 0.1435 µg/mL (Fig. 3D, middle left). Deletion of *orfH* resulted in a 15.3% decrease in the MIC, and deletion of *omp26* resulted in an insignificant decrease in the MIC at 7.2%, indicating that a loss of either of these genes had relatively little effect on the ability of Hi375 to resist CLR (Fig. 3D, middle left). In contrast, NT127 had the highest MIC of these three strains at 6.533 ± 0.1318 µg/mL, and the largest decreases in MIC when *orfH* and *omp26* were deleted at 52.6% and 33.8%, respectively (Fig. 3D, middle right), indicating that NT127 is more dependent on OrfH and Omp26 than the other two strains to survive during growth with CLR. Overall, the data indicate that there is a strain-specific increase in susceptibility to CLR when the outer membrane is altered by the loss of *orfH* or *omp26*.

**TABLE 1:** Calculated MIC values for strains in Fig. 3^*a*^

Strains	MIC (μg/mL)	CLSI designation
RdAW	4.992 ± 0.1665	Susceptible
RdΔorfH	3.391 ± 0.2783	Susceptible
RdΔomp26	3.743 ± 0.2158	Susceptible
Hi375	5.252 ± 0.2317	Susceptible
375ΔorfH	4.451 ± 0.1435	Susceptible
375Δomp26	4.875 ± 0.3444	Susceptible
NT127	6.533 ± 0.1318	Susceptible
NTΔorfH	3.097 ± 0.05782	Susceptible
NTΔomp26	4.324 ± 0.07626	Susceptible
AcrR′	10.72 ± 0.6569	Intermediate
AcrR′ΔorfH	6.132 ± 0.7068	Susceptible
AcrR′ΔorfH/V	6.298 ± 0.2287	Susceptible
AcrR′ΔorfH/C	8.449 ± 0.4584	Intermediate
AcrR′Δomp26	6.897 ± 0.3097	Susceptible
AcrR′Δomp26/V	6.020 ± 0.3638	Susceptible
AcrR′Δomp26/C	9.699 ± 0.8751	Intermediate

^*a*^ MIC was calculated using a modified Gompertz function (see Materials and Methods).

### Targeting *orfH* and *omp26* in an overexpressed efflux pump background

One of the genes identified to enhance fitness in the presence of CLR in our transposon library was *acrR*. Many CLR-resistant strains isolated from previously treated patients have a nonsense mutation in the *acrR* gene, which encodes the negative regulator AcrR of the multidrug efflux pump AcrAB (61). A nonsense mutation results in a nonfunctional truncated AcrR, leading to an excessive number of efflux pumps that can pump out a higher volume of CLR than wild-type strains. While an overexpressed efflux pump alone can increase the MIC by at least threefold, this mutation typically precedes missense mutations in ribosomal subunits, such as L22 or L4, that lead to an even stronger resistance phenotype (33, 67, 68). Due to the prevalence of an overexpressed efflux pump among CLR-resistant clinical isolates, we wanted to determine if targeting *orfH* and *omp26* in this background would confer sensitivity by overcoming an overexpressed efflux pump. We generated a strain in the Rd background that possesses a nonsense mutation commonly seen in CLR-resistant clinical isolates (denoted as AcrR′) (61). In this new background, we introduced in-frame deletions of *orfH* and *omp26* and used our modified MIC assays to determine the effect of these deletions on its sensitivity to CLR. We also constructed complemented strains for AcrR′ΔorfH and AcrR′Δomp26 by expressing *orfH* or *omp26* under the control of a xylose-inducible promoter at the *xyl* locus (Materials and Methods).

The MIC for the non-susceptible AcrR′ strain was 10.720 ± 0.6569 µg/mL CLR, making this strain twofold more resistant than the wild type (Fig. 3D, far right and Table 1). Deletion of *orfH* resulted in a decrease of 42.8% in the MIC. The empty vector strain AcrR′ΔorfH/V had a similar decrease at 41.2%, while partial restoration is seen in the complemented strain AcrR′ΔorfH/C with only a 21.2% drop (Fig. 3D, far right). AcrR′Δomp26 exhibited a 35.7% decrease in the MIC, and its empty vector derivative AcrR′Δomp26/V showed a 43.8% decrease in the MIC. When *omp26* was complemented, a strong restoration of resistance was seen, with only a 9.5% decrease in the MIC compared to AcrR′ (Fig. 3D, far right). Based on these results, we conclude that removal of *orfH* and *omp26* abrogated nearly all resistance conferred by the truncated AcrR, indicating that an overexpressed efflux pump is not sufficient to maintain resistance when outer membrane components have been altered.

### Mechanism of increased sensitivity of mutants in the AcrR′ background

Because OrfH and Omp26 both play a role in maintaining the integrity of the outer membrane, we hypothesized that the removal of these genes resulted in increased permeability in the deletion strains and caused an increase in susceptibility to CLR. To evaluate this hypothesis, we performed ethidium bromide accumulation fluorescence assays. Because ethidium bromide is a substrate of AcrB (62, 69), we can compare relative measurements of accumulation between strains based on permeability while taking the overexpression of AcrAB into consideration.

**Fig 4:**
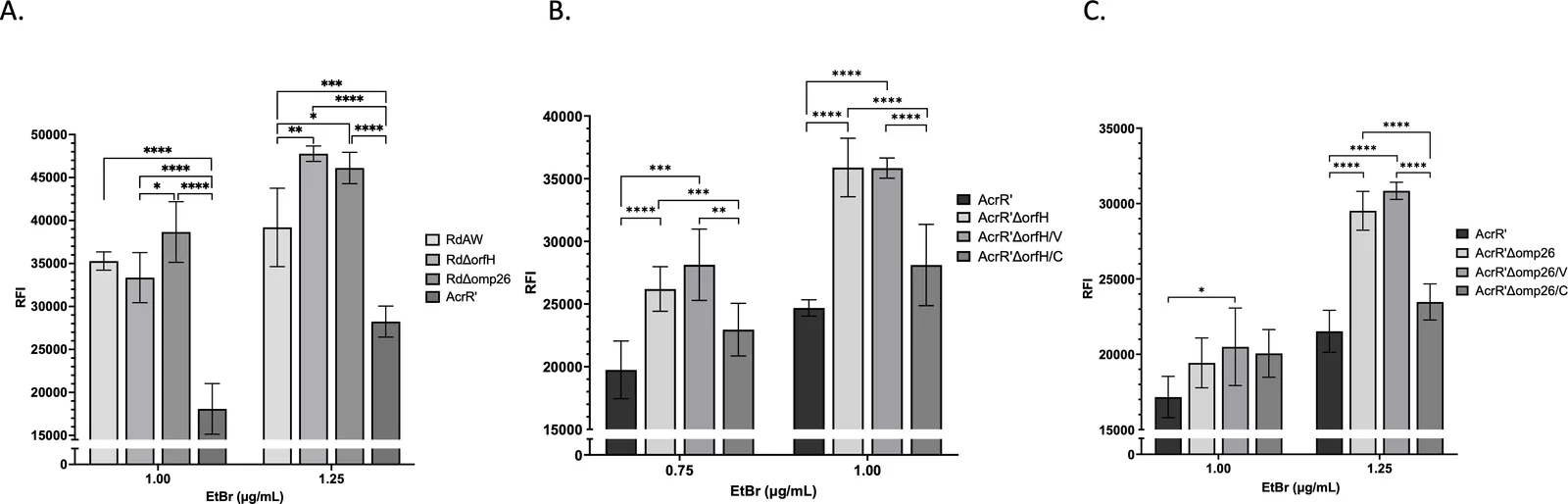
Permeability was increased in the *orfH* and *omp26* deletion mutants. Each strain was grown to mid-log phase and incubated with ethidium bromide for 30 min before fluorescence was measured for (**A**) Wildtype RdAW versus mutants, (**B**) Rd AcrR’ versus *orfH* deletion mutants and complemented strain, and (**C**) Rd AcrR’ versus *omp26* deletion mutants and complemented strain. Results are shown as the mean of duplicate samples. Differences were evaluated via one-way ANOVA with Tukey’s multiple comparisons test (****, *P* < 0.0001; ***, *P* < 0.001; **, *P* < 0.01; and *, *P* < 0.05).

In the RdAW background, the AcrR′ strain accumulated the least amount of ethidium bromide due to the overexpressed efflux pump with a fully intact outer membrane (Fig. 4A). At lower levels of ethidium bromide, RdAW and deletion mutants lacking *orfH* or *omp26* had comparable accumulation; however, at higher levels, the deletion mutants accumulate more than the wild type (Fig. 4A). In the AcrR′ background, AcrR′ΔorfH and the empty vector AcrR′ΔorfH/V strain accumulated more ethidium bromide than AcrR′ and the complemented AcrR′ΔorfH/C strain (Fig. 4B). The same phenomenon is seen for *Δomp26* in the AcrR′ background. AcrR′Δomp26 and AcrR′Δomp26/V accumulated more ethidium bromide than AcrR′ and AcrR′Δomp26/C at higher concentrations of ethidium bromide (Fig. 4C). At lower concentrations, all four strains accumulated roughly equal amounts although accumulation in the AcrR′ strain decreased by a non-statistically significant amount (Fig. 4C). The results indicate that deletions of *orfH* and *omp26* lead to increased permeability in these mutants, correlating with increased sensitivity to CLR.

### Requirement for Omp26 in serum resistance

Next, we wanted to determine the contribution of Omp26 to infection. We identified *omp26* as being necessary for infection in our HITS experiment involving the mouse lung model, but the mechanism of its contribution to infection, to our knowledge, is unknown. Since OrfH has previously been confirmed to be necessary for infection via resistance to the human complement system (40, 55), we sought to determine whether Omp26 also might play a role in serum resistance. To begin investigating the mechanism of Omp26 in infection, we examined the *E. coli* periplasmic chaperone Skp, in which Omp26 shares an overall 32% amino acid sequence identity (53). Aligning the predicted structures of Omp26 and Skp reveals a TM-score of 0.71 (Fig. S1), indicating a structural similarity that suggests that Omp26 may function in a similar manner (70–74). As a periplasmic chaperone, Omp26 would be responsible for the proper incorporation into the outer membrane of proteins that might play a role in resisting human complement. To determine the necessity of Omp26 in *H. influenzae* for serum resistance, we performed serum bactericidal assays using normal human serum (NHS).

**Fig 5:**
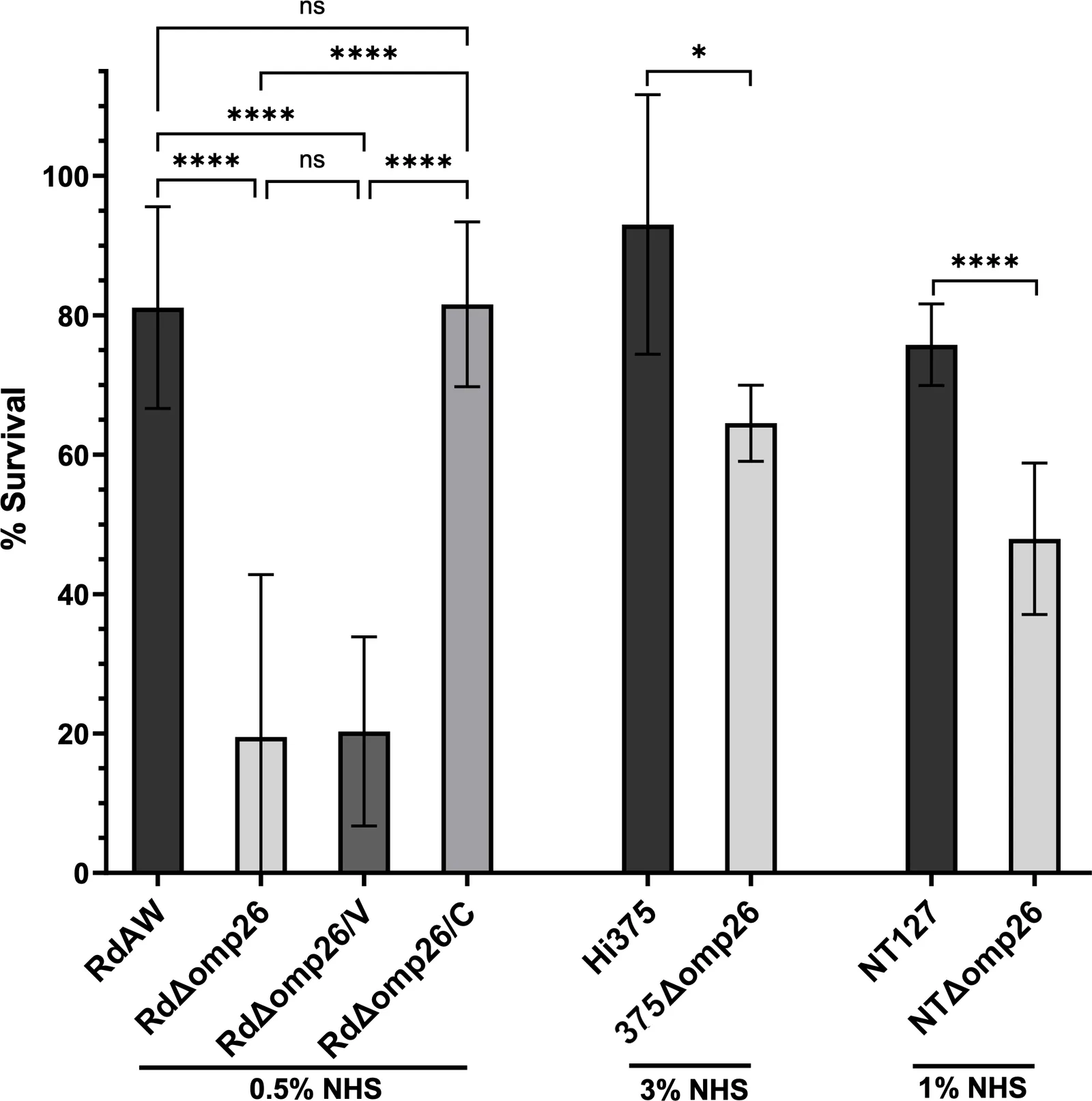
Omp26 is necessary for resistance to human serum. Strains were grown to mid-log phase and incubated with the indicated concentration of normal human serum. Percent survival was calculated as the ratio of the number of colonies recovered from samples incubated with NHS to the number of colonies recovered from samples incubated with heat-inactivated NHS (NHS^Δi^). The mean of triplicate samples is shown. Differences were evaluated via one-way ANOVA with Tukey’s multiple comparisons test (****, *P* < 0.0001; *, *P* < 0.05; and ns, not statistically significant).

At 0.5% NHS, RdAW and RdΔomp26/C survival rates were similar at ~80%. When *omp26* was deleted, the percent survival decreased to ~20% for both RdΔomp26 and RdΔomp26/V (Fig. 5, left). Both nontypeable strains, Hi375 and NT127, also failed to maintain wild-type levels of resistance to human serum in the absence of *omp26*. The survival rate for Hi375 fell from ~95% to ~65% (Fig. 5, middle), while NT127 exhibited a decrease from ~80% to ~50% (Fig. 5, right). The data indicate that all three strains rely on Omp26 for resistance to the human complement system.

### Sub-MIC CLR treatment effect on polymyxin B resistance

**Fig 6:**
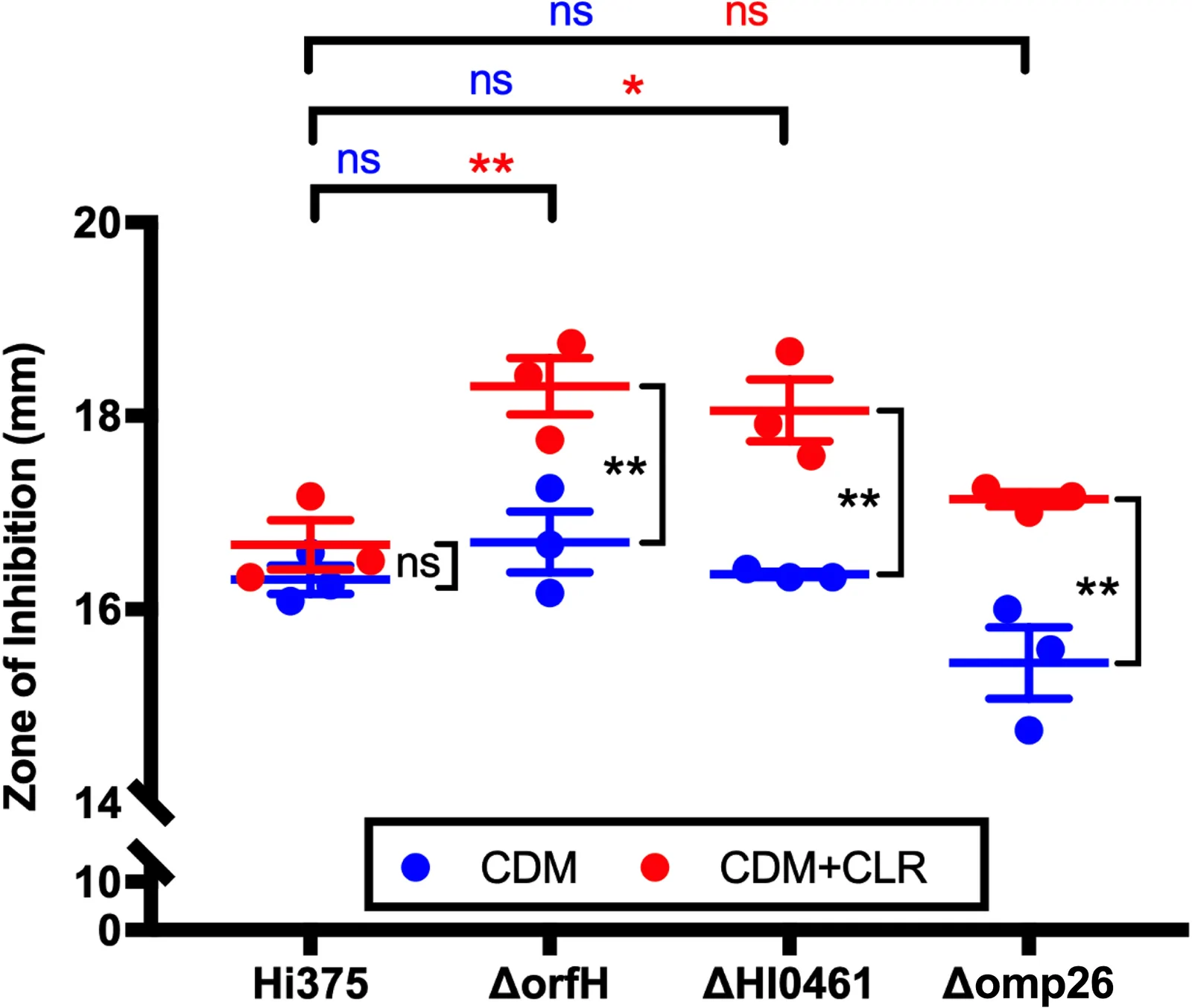
Pretreatment with clarithromycin increased sensitivity of Hi375 deletion mutants to polymyxin B. *H. influenzae* Hi375 and its indicated isogenic mutants were grown on either CDM or CDM + sub-MIC CLR (CDM + CLR) plates with disks containing 0.5 mg/mL polymyxin B. Zones of inhibition were measured after overnight incubation. Differences were evaluated via ANOVA with Šidák’s multiple comparisons test (*, *P* < 0.05; **, *P* < 0.005; and ns, not statistically significant).

Because exposure to sub-MIC antibiotics can alter bacterial susceptibility to subsequent treatment with antibiotics (75), we examined how CLR treatment might affect the susceptibility of our strains to polymyxin B, a bacterially produced antimicrobial peptide that shares the amphipathic nature of immune system-produced antimicrobial peptides. Like host-generated antimicrobial peptides, polymyxin B interacts with the negative charges on the bacterial outer membrane, resulting in disruption of the outer membrane and bacterial lysis (76, 77). Therefore, we used it as a model for antimicrobial peptides. To test if treatment with CLR could alter susceptibility to polymyxin B, we grew strains overnight on CDM agar with or without sub-MIC CLR and then performed disk diffusion assays with polymyxin B disks on the same media used to grow each overnight culture. Sub-MIC treatment with CLR did not affect the sensitivity of the wild-type Hi375 strain (Fig. 6). CLR treatment increased polymyxin B sensitivity of all deletion mutants compared to lack of treatment for each strain; moreover, the *orfH* and HI0461 mutations showed increased sensitivity to CLR treatment compared to the parent strain Hi375. The *orfH* and HI0461 mutants in Rd similarly showed increased polymyxin B sensitivity (Fig. S2). Our previous studies have validated the requirement for *orfH* by *H. influenzae* NT127 in the murine lung model (40). Therefore, our current results indicate that in addition to complement, antimicrobial peptides may apply added *in vivo* selective pressure on *H. influenzae* lacking these genes and thereby work synergistically with CLR.

## DISCUSSION

Macrolide antibiotics, which inhibit protein synthesis and ribosome assembly, are often used to treat *H. influenzae* infections, particularly respiratory tract infections. However, *H. influenzae* can intrinsically resist macrolide treatment due to the presence of its multidrug efflux pump system *acrAB*. Resistance to macrolide antibiotics such as CLR can further be acquired through deregulation of *acrAB* and mutations in the ribosome. In other systems such as *Acinetobacter baumannii* (78), the level of intrinsic resistance to antibiotics such as tobramycin depends on multiple genes working together, and we anticipate multiple gene involvement for intrinsic resistance to CLR in *H. influenzae* as well. Intrinsic macrolide resistance in *H. influenzae* has not yet been explored on a genome-wide level to date. In this report, we used our transposon mutant screening technique (HITS) on a mutant library in *H. influenzae* at two sub-inhibitory doses of CLR to identify 33 genes involved in CLR resistance: 30 that are needed for resistance and 3 that showed increased relative fitness in the presence of CLR. Many encode novel resistance proteins such as the ribosomal protein L9 and the vaccine candidate HI0916 (OMP26) (Fig. 1). Our pan-genome analyses indicate that most of these genes contributing to CLR resistance are conserved and present in the core genome of *H. influenzae*. It is intriguing that almost half of the genes needed for CLR resistance are also required for survival in our mouse lung model (Fig. 1), suggesting that the effects of CLR may reflect aspects of the lung environment.

Identification of genes involved in both antibiotic resistance and host resistance mechanisms suggests an overlap in their functions related to these phenotypes. Such is the case in *Streptococcus pneumoniae*, where treatment with human antimicrobial peptide LL-37 induced resistance to both erythromycin and LL-37 via induction of expression of the efflux pump MefE/Mel (79). Reports by others have identified *in vivo* virulence genes that have also been previously implicated in antibiotic resistance (80). Our data identified genes involved in fitness in the lung and in the presence of CLR that were not previously known to be involved in CLR resistance. Overall, these results indicate that numerous genes required for intrinsic resistance to antibiotics play multiple functional roles, including survival and immune evasion during infection. Therefore, our results suggest that components of the immune system may target or otherwise generate survival requirements for some of the CLR resistance gene products.

For validation of the genetic screen, we confirmed the CLR sensitivity profile of three genes needed for intrinsic CLR resistance, *orfH*, *omp26*, and *HI0461,* in three wild-type *H. influenzae* strain backgrounds, Rd and NTHi strains Hi375 and NT127 (Fig. 2 and 3; Table S7). These genes are known or suspected to affect the surface structure or the outer membrane of this bacterium (51, 52), a key structural factor in intrinsic resistance, and have not previously been shown to be involved in CLR resistance. The importance of the cell surface and its components to intrinsic resistance to antibiotics that do not target synthesis of the cell wall has been investigated in *Mycobacterium tuberculosis,* in which genes affecting the cell envelope permeability were found to be major determinants of intrinsic antibiotic resistance (81). Therefore, insights into the dual phenotypes of genes affecting the outer membrane may relate to additional pathogens.

We showed that the *orfH* and *omp26* genes are also needed for CLR resistance in Rd strain AcrR′, which becomes antibiotic resistant via a mutation created in *acrR* in this study (Fig. 3, Materials and Methods). Consistent with the CLR sensitivity results, *orfH* and *omp26* mutants were also more membrane permeable to ethidium bromide in both wild-type Rd and AcrR′ backgrounds (Fig. 4). This indicates that deletion of these genes not only mitigates but can also override the effects of an acquired resistance mechanism. This has significance, as the presence of a mutation found in *acrR* commonly causes increased drug efflux in antibiotic-resistant clinical isolates, not only in *H. influenzae* (33, 34) but also in other pathogens as well (82, 83).

For genes affecting the outer membrane, CLR resistance to sub-inhibitory doses may functionally overlap with resistance to host defense mechanisms in the lung that act on the outer membrane, possibly through antimicrobial activity of innate immune system components such as the complement system and antimicrobial peptides. Thus, we examined the role of serum complement in killing the *omp26* deletion mutants (Fig. 5) and the role of polymyxin B (a bacterial peptide whose activity against pathogens strongly correlates with that of host-derived antimicrobial peptides) in inhibiting the growth of the *orfH*, *omp26*, and *HI0461* deletion mutants (Fig. 6; Fig. S2). Both *orfH* and *HI0461* were implicated in polymyxin B resistance in the presence of sub-MIC CLR, and we show a newly identified role for *omp26* in resisting killing by human serum (Fig. 5). Understanding mechanisms of the dual roles of these genes in intrinsic resistance and host-pathogen interactions may potentially aid in developing screens for novel antimicrobials that could synergize with CLR, likely with a reduced frequency of escape mutations, as these genes are also required for immune evasion.

Deletion of *orfH* and *omp26* had strain-dependent effects on *H. influenzae* that may arise from structural differences in the LOS or differences in expression or structure of outer membrane proteins. In the three *H*. *influenzae* strains studied here, deletion of *orfH* or *omp26* had the greatest effect on NT127 and the least effect on Hi375 among the wild-type backgrounds (Fig. 3). In the AcrR′ background, deletion of *orfH* or *omp26* had greater effects on its ability to resist CLR than in the wild-type Rd background. In the case of OrfH, which adds the final third heptose group to the inner core of the LOS, the differences in affecting intrinsic antibiotic resistance may pertain to the extensions on LOS. All three strains used here have identical inner cores but differ in their outer core. NT127 has its outer core extensions on heptose II, rather than the extensions on heptose III observed in RdAW and Hi375 (Fig. 3A through C). Failure to add heptose III might result in a loss of stabilization of the LOS structures that could allow for greater penetration of CLR (84) and may explain why the deletion of *orfH* had a stronger effect in this strain than in RdAW and Hi375.

LOS extensions are not the only source of variability among NTHi strains. Although these strains express many of the same major outer membrane proteins, allelic variation in sequences of genes encoding outer membrane proteins is extensive among nontypeable strains (85, 86). Thus, it could explain why the effect of deleting *omp26* on CLR resistance was strain-dependent. Interestingly, effects of deletions of *omp26* followed the same pattern of strain dependency for CLR resistance among these three strains as seen for *orfH*, possibly indicating that the outer membranes of RdAW and Hi375 may be inherently more stable than that of NT127.

From our genetic screen, we identified genes that have not previously been implicated in macrolide CLR resistance, such as ribosomal protein L9, which functions in protein translation (Fig. 1), expanding on reports that L4 and L22 mutations enhanced resistance to macrolides (67, 68). As part of the macrolide mechanism of action, CLR binds within the nascent peptide exit tunnel (NPET), close to the peptidyl transferase center (PTC), to halt protein synthesis (87). L4 lies near the PTC, while L22 helps form the NPET. Mutations in these proteins prevent macrolides from binding to their target by changing the conformation of the NPET and preventing macrolides’ access. In contrast, L9 does not lie near either the NPET or the PTC, and yet it maintains translation fidelity and prevents frameshifting (88). Interestingly, one study found that L9 was necessary for growth in the presence of antibiotics that cause miscoding (88), and in another study, up to 6% of *E. coli*’s proteome continued to be synthesized in the presence of erythromycin and up to 25% in the presence of telithromycin, a next-generation macrolide of the ketolide class (87). Macrolides can induce frameshift mutations (89), and some proteins can escape macrolide-inhibited protein synthesis (87). We hypothesize that L9 might play a role in maintaining translational fidelity in the presence of CLR.

Our HITS experiment provided another insight, implicating several genes associated with oxidative stress that may play a role in CLR activity against *H. influenzae*. Oxidative stress is involved in bactericidal antibiotic killing (90, 91) but not in all cases (92, 93). One study showed that protein synthesis-halting antibiotics like aminoglycosides generated reactive oxygen species (ROS) that contributed to killing (91). In support of our findings, Smith et al. (94) demonstrated that CLR killing is greater against *H. influenzae* in aerated cultures compared to microaerophilic cultures grown in 5% CO_2_. Several oxidative stress genes that were identified in our study were part of the methionine uptake system (*metN* and *metI*), methionine biosynthesis pathway (*metE*), or regulation of the methionine regulon (*metJ*). *H. influenzae* may be relieving oxidative stress during CLR treatment by using methionine as an antioxidant (95). Additionally, *recA*, known to repair ROS-mediated DNA damage (96), was identified to be required at the higher CLR concentration.

Continuous use of macrolides to treat COPD and its recurring exacerbations leads to resistance that develops in NTHi-infected individuals (27). Our HITS analysis identified *acrR*, a clinically relevant gene known to contribute to macrolide resistance in isolates found in COPD patients over the course of treatment. In a study by Tsuji et al. (27), the long-term effect of azithromycin usage on *H. influenzae* persistence was examined. Strain 5P28H1, inferred to be the parent strain, was isolated at the beginning of the study prior to azithromycin treatment. However, strain 5P54H1 isolated at the end of the study had a substitution of glycine to aspartic acid at position 91 in the ribosomal protein L22, one of the most common mutations seen in macrolide-resistant clinical isolates (34, 67, 68). Interestingly, our pan-genome analysis identified a frameshift mutation in *acrR* in 5P54H1, while the parent 5P28H1 had no mutations in this gene. Moreover, in clinical isolates, mutations in *acrR* are seen not only in conjunction with substitutions in L22 but also in L4, AcrAB, and 23S rRNA (34). Together, the L22 and *acrR* mutations could contribute to enhanced azithromycin resistance.

Genes that are required for CLR resistance and survival in the murine lung infection model represent promising target candidates for novel antimicrobial strategies. Targeting the product of one of these genes alongside CLR usage would be able to address current resistance problems by (i) making these isolates more susceptible to CLR by targeting a specific protein necessary for intrinsic resistance; (ii) making all isolates susceptible to clearance by the host by targeting a protein necessary for surviving within the host; and (iii) reducing future evasion by targeting a protein whose function is essential for infection to constrain potential escape mutations. Overall, we identified novel intrinsic resistance genes for *H. influenzae* that are involved in cell wall and outer membrane biogenesis and modification, oxidative stress, transport/efflux, transcription, and translation. Moreover, we identified a large subset of intrinsic CLR resistance determinants that are also involved in virulence phenotypes, including complement resistance. Future studies are required to define specific mechanisms of immune evasion mediated by these genes and to extend these studies to macrolide-resistant clinical isolates. Ultimately, these data may aid in the design of new combination therapies to be utilized with macrolides.

## MATERIALS AND METHODS

### Bacterial strains and growth conditions

The non-encapsulated *H. influenzae* type d RdAW and nontypeable *H. influenzae* clinical isolates Hi375 and NT127 were grown at 35°C with 5% CO_2_. The medium used was brain heart infusion supplemented with 10 µg/mL nicotinamide adenine dinucleotide (NAD) and 10 µg/mL hemin (sBHI) on agar plates or Mueller-Hinton broth supplemented with 15 μg/mL of NAD and hemin, and 5 mg/mL yeast extract (sMH) for routine growth as indicated. sBHI and sMH broths used for routine growth for experiments in which complemented strains were included were further supplemented with 0.02% xylose (~1 mM) for inducible expression of the complementing copy of wild-type genes under the *xylA* promoter in pXT10 (97). For deletion mutant strain construction, strains were grown on sBHI with 15 µg/mL gentamicin or 20 µg/mL kanamycin supplementation as indicated. Chemically defined medium was prepared as described by Herriott et al. (44), using the MIC formulation with slight modifications. Sub-MIC CLR plates were CDM plates supplemented with 0.75 µg/mL CLR. Naturally competent *H. influenzae* was prepared for DNA transformation as described (98). Competent strains were plated on LacZ indicator agar, which consisted of sBHI supplemented with 0.488 mg/mL D-xylose, 0.26 mg/mL 3,4-cyclohexenoesculetin-β-D-galactopyranoside (S-gal), and 0.5 mg/mL ferric ammonium sulfate.

### Mutant library *in vitro* growth

The *H. influenzae* Rd transposon mutant library was grown in sBHI at 35°C with shaking at 150 rpm until it reached an optical density at 600 nm (OD_600_) of ~0.3–0.4 and was inoculated into CDM at ~10^7^ CFU/mL in Hank’s balanced salt solution containing calcium and magnesium chloride (HBSS++). Aliquots of the mutant library in HBSS++, representing the input, were frozen at –80°C. *H. influenzae* was grown for 20 h in CDM with either no supplementation, supplemented with the solvent as a control (5% DMSO and 0.5% methylcellulose to dissolve CLR), or 12.5, 6.25, 3.13, 1.56, or 0.78 µg/mL CLR. Then, OD_600_ values were measured in a spectrophotometer (Bio-Rad). The minimum inhibitory dose of CLR was 12.5 µg/mL; thus, the mutant libraries were grown in the presence of 6.25 µg/mL (high) and 3.13 µg/mL (low) CLR, doses that would not kill intrinsically resistant mutants, and were processed for HITS analysis. The mutant libraries were pelleted, resuspended in TEN (10 mM Tris [pH 8.0], 5 mM EDTA, and 100 mM NaCl) buffer, and frozen at –80°C.

### Murine lung infections with mutant library

The *H. influenzae* Rd transposon mutant library was grown in sBHI at 35°C with shaking at 150 rpm until it reached an OD_600_ of ~0.3–0.4, pelleted, and resuspended in HBSS++ to the appropriate cell concentration for infection. Mutant library (~ 10^7^ CFU/mL) in 40 µL of HBSS++ was inoculated intranasally into 6- to 8-week-old C57BL/6 mice. After 22 h of infection, mice were euthanized; lungs were harvested, homogenized, and then plated for CFU determination.

### Mutant library DNA preparation for sequencing

Frozen mutant libraries from murine lung homogenates were thawed, pelleted, and resuspended in 450 µL of TEN buffer. One 450 µL aliquot of each of the frozen mutant libraries from CDM ± CLR was thawed. Then, 50 µL of 20 mg/mL proteinase K and 30 µL of 20% SDS were added to the mutant libraries in TEN buffer and incubated at 55°C for 30 min. Genomic DNA was isolated by phenol extraction, followed by phenol/chloroform extraction and ethanol precipitation. gDNA was treated with 200 µg/mL RNase A and then re-isolated by phenol/chloroform extraction and ethanol precipitation. gDNA was resuspended in sterile ddH_2_O with the concentration, and A_260_/A_280_ was determined using a Nanodrop 2000 (Thermo Scientific). To prepare the gDNA for sequencing, the gDNA was sheared by a Covaris S2 instrument to a target size of ~300 bp and then concentrated using a Qiagen PCR purification column, end repaired, an “A” base was added to the 3′ ends, and adapters (adap1 and adap3) were ligated (adapter sequences in Text S1) as described (97). Then, two nested PCR reactions using the Phusion High-Fidelity PCR kit (Thermo Scientific) were performed (primer sequences in Text S1) to enrich for the transposon-chromosomal junctions (PCR 1; primers: marout-nest 2 and oljJD) and add Illumina primer capture sites (PCR 2; primers: MarPCR2-5N and BC#). The first PCR ran for 19 cycles and was purified using a Qiagen PCR purification column, and the second PCR ran for 5 cycles and was purified by Ampure XP (Beckman Coulter). Final library concentrations were measured using an Invitrogen Qubit Fluorometer 2.0.

### Analysis of sequencing data

Sequencing reads from FASTQ files were mapped, and transposon insertion sites were counted using the transposon insertion site sequencing analysis pipeline Tn-Seq Pre-Processor (99). Then, essentiality was determined using the pipeline TRANSIT. Individual gene essentiality was determined using the hidden Markov model analysis in which genes were called as essential, non-essential, growth defective, or growth advantaged. Conditional gene essentiality was determined using the resampling analysis in which all of the data sets that were involved in each individual conditional analysis (e.g., input replicates and CDM replicates) were resampled to evaluate the significance of the differences. Resampling significance cutoffs were ≥2- and ≤−2-fold change and an adjusted *P* value < 0.05, which were adjusted for multiple comparisons using the Benjamini-Hochberg procedure.

### Pan-genome analysis

The pan-genome and core genome were determined using the pan-genome analysis pipeline Roary (https://sanger-pathogens.github.io/Roary/) (59). One hundred twelve complete and scaffold genomes that had protein annotation information were downloaded from NCBI (Table S4). Using the Bio::Perl script bp_genbank2gff3.pl, the gbff files were converted to GFF3 files as described (https://sanger-pathogens.github.io/Roary/). The 112 converted files were used for the pan-genome analysis through the Roary pipeline. Roary was also run with 110 genomes, excluding genomes 5P54H1 and 17, because of a discrepancy for *acrR* (HI0893) in the analysis with 112 genomes, which is explained in Text S1. Roary was run with a 90% blastp percent identity cutoff. Five pan-genomes were generated, and the mode was used to represent the number of genomes each gene appears in (Tables S5 and S6).

### Plasmid and *H. influenzae* strain construction

All primer sequences are listed in Text S1. Strains and plasmids used in the main studies are listed in Table 2. Standard molecular biology methods were used for PCR, cloning, and plasmid construction (100). The HI locus tag nomenclature is used for *H. influenzae* Rd KW20 genes assigned by TIGR (101).

**TABLE 2:** Strains and plasmids

	Genotype and/or relevant features	Reference
Strains		
RdAW	*H. influenzae*; wild type; nonencapsulated serotype d	(40)
RdΔomp26	RdAW Δomp26::*aaC1*; Gm^r^	This study
RdΔomp26/V	RdΔomp26 with empty pXT10 at the *xyl* locus; Gm^r^; Tet^r^	This study
RdΔomp26/C	RdΔomp26 with *omp26* expressed under the xylose-inducible promoter for complementation at the *xyl* locus; Gm^r^; Tet^r^	This study
RdΔorfH	RdAW ΔorfH::*aphI*; Km^r^	This study
AcrR′ (Resistant)	RdAW with a single nucleotide deletion at nucleotide 141 in *acrR* to induce a frameshift that results in a truncated AcrR	This study
AcrR′ΔorfH	AcrR’ ΔorfH::*aphI*; Km^r^	This study
AcrR′ΔorfH/V	AcrR’ΔorfH with empty pXT10 at the *xyl* locus; Km^r^; Tet^r^	This study
AcrR′ΔorfH/C	AcrR’ΔorfH with *orfH* expressed under the xylose-inducible promoter for complementation at the *xyl* locus; Km^r^; Tet^r^	This study
AcrR′Δomp26	AcrR’ Δomp26::*aac1*; Gm^r^	This study
AcrR′Δomp26/V	AcrR’Δomp26 with empty pXT10 at the *xyl* locus; Gm^r^; Tet^r^	This study
AcrR′Δomp26/C	AcrR’Δomp26 with *omp26* expressed under the xylose-inducible promoter for complementation at the *xyl* locus; Gm^r^; Tet^r^	This study
Hi375	*H. influenzae*; wild type; nontypeable; clinical isolate	(65)
375ΔorfH	Hi375 ΔorfH::*aphI*; Km^r^	This study
375Δomp26	Hi375 Δomp26::*aac1*; Gm^r^	This study
375Δ0461	Hi375 Δ0461::*aac1*; Gm^r^	This study
NT127	*H. influenzae*; wild type; nontypeable; clinical isolate	(40)
NTΔorfH	NT127 ΔorfH::*aphI*; Km^r^	(40)
NTΔomp26	NT127 Δomp26::*aac1*; Km^r^	This study
Plasmids		
pXT10	Delivery vector for chromosomal expression at the *xyl* locus of *H. influenzae*, contains *xylF*, *xylB*, *xylA*^Δ4-802^, and *tetAR* tetracycline resistance cassette; Tet^r^; referred to as /V	(102)
pXRorfH	pXT10 with wild-type RdAW copy of *orfH* expressed under the *xylA* promoter; referred to as /C	This study
pXRomp26	pXT10 with wild-type RdAW copy of *omp26* expressed under the *xylA* promoter; referred to as /C	This study

### Construction of in-frame deletion strains

Nonpolar, in-frame deletion mutations were created in the genes HI0461, HI0916 (*omp26*), and HI0523 (*orfH*) by overlap PCR stitching. A gentamicin resistance cassette (*aacC1*) replaced HI0461 and HI0916 in RdAW and Hi375. The 5′ and 3′ flanking regions for each gene were amplified with primers containing complementary overlapping ends to the gentamicin resistance cassette. The gentamicin resistance cassette was amplified with primers 5Gent2 and 3Gent2, generating a 534-bp product. For HI0916, the 5′ flanking region was amplified with primers 5′-HI0916-omp26 and 3′-HI0916-omp26-gen, generating a 576-bp product. The 3′ flanking region of HI0916 was amplified with primers 5′-HI0916-omp26-gen and 3′-HI0916-omp26, generating a 566-bp product. Then, the 5′-HI0916-omp26 and 3′-HI0916-omp26 primers were used to stitch the gentamicin resistance gene and the 5′ and 3′ flanking regions of HI0916, generating a 1,676-bp product. For HI0461, the 5′ flanking region was amplified with primers 5_HI0461_1 and 3_HI0461_1g, generating a 561-bp product. The 3′ flanking region of HI0461 was amplified with primers 5_HI0461_2g and 3_HI0461_2, generating a 647-bp product. Then, the 5_HI0461_1 and 3_HI0461_2 primers were used to stitch the gentamicin resistance gene and the 5′ and 3′ flanking regions of HI0461, generating a 1,738-bp product. Naturally competent *H. influenzae* Rd and Hi375 cells were transformed with stitched PCR products, and transformants were selected on sBHI agar containing 15 µg/mL gentamicin. Deletions were verified by PCR amplification with the following primers: 5′-HI0916-out and 3′-HI0916-out for ∆HI0916 and 5′-HI0461-out and 3′-HI0461-out for ∆HI0461, which flank the recombinant region of the deletions. For NT127, HI0916 was replaced with the kanamycin resistance cassette (*aphI*), which was amplified with primers 5Kan1.1 and 3Kan1.1, generating an 816-bp product. The 5′ flanking region was amplified with primers 5-HI0916-omp26 and 3-HI0916-Kan, generating a 591-bp product. The 3′ flanking region was amplified with primers 3-HI0916-omp26 and 5-HI0916-Kan, generating a 596-bp product. The 5′ and 3′ flanking regions were stitched to the kanamycin cassette with primers 5-HI0916-omp26 and 3-HI0916-omp26, generating a 1,955-bp product. NT127 cells were transformed with stitched PCR products, and transformants were selected on sBHI agar containing 20 µg/mL kanamycin. Deletion was verified by PCR amplification with primers 5′-HI0916-out and 3′-HI0916-out. Deletion of *orfH* in NT127 (creating strain NT∆orfH) is described previously (40). Using NT∆orfH as a template, primers HI0524-int5′ and HI0521-int3′ were used to amplify a 3.0 kb PCR product containing 5′ and 3′ regions flanking the deletion of *orfH*. The product was introduced into naturally competent *H. influenzae* RdAW and Hi375 cells, and transformants were selected on sBHI agar containing 20 µg/mL kanamycin. Deletions were verified by PCR analysis with primers HI0524-5′-orf and HI0521-3′-orf.

### Construction of *acrR* point deletion mutant and its isogenic deletion mutants

Nucleotide 141 in *acrR* (HI0893) was deleted in RdAW to generate a CLR-resistant strain, RdAW AcrR′ as follows. The 5′ portion of *acrR* was amplified with primers acrR_F and acrR_d141nt_R, containing the point deletion, generating a 796-bp product. The 3′ portion was amplified with primers acrR_R and acrR_d141nt_F, which also contained the point deletion, generating a 680-bp product. The two fragments were stitched together with primers acrR_F and acrR_R, generating a 1,457-bp product. Naturally competent RdAW cells were transformed with the stitched PCR product, and transformants were selected on sBHI agar containing 10 μg/mL CLR and verified with Sanger sequencing. In-frame deletion of genes HI0916 (*omp26*) or HI0523 (*orfH*) was then created in RdAW AcrR′. Cells were transformed with stitched PCR products, and transformants were selected on sBHI agar containing 20 µg/mL gentamicin (HI0916) or 25 µg/mL kanamycin (HI0523). Deletions were verified by PCR analysis with primer pairs 5′-HI0916-out and 3′-HI0916-out for HI0916 and HI0524-5′-orf and HI0521-3′-orf for HI0523.

### Mutant complementation

For complementation of *orfH* and *omp26*, the allelic exchange vector pXT10 was used to deliver wild-type genes into the *xyl* locus (102). HI0523 (*orfH*) was amplified from RdAW genomic DNA with 3-HI0523-SapI and 5-HI0523-SapI, generating a 1,109-bp product. HI0916 (*omp26*) was amplified from RdAW genomic DNA with 3-HI0916-SapI and 5-HI0916-SapI, generating a 627-bp product. Each of these resultant products was cloned into pXT10 to create pXRorfH and pXRomp26, respectively. These plasmids were linearized with PciI and SacI and transformed into naturally competent *H. influenzae* RdAW and RdAW AcrR′ *omp26* deletion mutants or RdAW AcrR′ *orfH* deletion mutant as previously described (98). Transformants were selected on sBHI agar containing 2 µg/mL tetracycline. Insertions were verified by PCR and Sanger sequencing. Deletion mutants that are complemented or containing the empty pXT10 vector are referred to as /C and /V, respectively.

### MIC determination

*H. influenzae* strains were grown overnight on CDM plates, harvested in HBSS++, and adjusted to an OD_600_ of 0.001 in CDM. Cells were seeded in 96-well plates, and CLR was applied in twofold dilutions, with three to six replicates of each strain. Cells were grown overnight, and the MIC was determined to be the lowest concentration with no growth. All strains had a standard error of the mean of 0 except 375∆omp26, which was 0.659.

### Competition assay

A *H. influenzae* strain (RdlacZ) expressing *lacZ* ectopically in the *xyl* locus as previously described (103) was used to compete with Rd and Rd∆omp26. The strains were grown overnight on CDM and swabbed into HBSS++. Then, the optical density of each strain was adjusted to an OD_600_ of 0.01 in CDM with and without sub-MIC (1.56 µg/mL) CLM, grown for 5 and 22 h, and then plated onto sBHI with D-xylose, 3,4-cyclohexenoesculetin-β-D-galactopyranoside (S-gal), and ferric ammonium sulfate. The RdlacZ strain appeared as black colonies, and Rd and Rd∆omp26 appeared as white colonies. The colonies were counted after ~24 h of growth, and the ratios between RdlacZ and Rd, RdlacZ and Rd∆omp26 were calculated, with three replicates of each competition.

### MIC microbroth assay with calculated MIC

*Haemophilus influenzae* strains were grown overnight in sMH. Cells were standardized to an OD of 0.001 (approximately 3.2 × 10^5^ CFU/mL of cells were seeded in each well) in sMH. CLR was applied to the wells in 0.8-fold serial dilutions. The plates were incubated overnight for 17–18 h at 36°C with 5% CO_2_. The results were read at 600 nm in a plate reader for endpoint OD_600_ with duplicates for each strain. Based on Lambert and Pearson (60) with minor modifications, the OD_600_ was plotted against the concentrations of CLR. The curve was fitted in GraphPad Prism using the modified Gompertz function (a), and the MIC value was calculated using values obtained from the resulting curve (b).

(a) *Y* = Bottom + Span × exp {−1 × exp[Slope × (*X* − *M)]}.*

(b) MIC = *M* + 1/Slope.

### Ethidium bromide accumulation

Ethidium bromide enters cells through passive diffusion and intercalates between DNA base pairs, which results in red-orange fluorescence under ultraviolet light (104) and allows for a relative measurement of accumulation within cells. Bacteria were grown to mid-log phase in sBHI, washed, and resuspended in DPBS at 0.7 OD. Ethidium bromide was applied to the cells at the indicated concentration and incubated at room temperature for 1 h. Next, cells were pelleted at 12,000 × *g* and washed again with DPBS. A volume of 200 μL of each sample was applied to a 96-well plate with duplicates of each strain. Fluorescence was measured at an excitation wavelength of 302 nm and an emission wavelength of 590 nm with a Bio-Rad gel imager. Fluorescence was analyzed with Bio-Rad Image Lab software.

### Serum bactericidal assays

*H. influenzae* strains were grown to mid-log phase in sMH, washed, and resuspended in HBSS++ supplemented with 0.1% bovine serum albumin. Bacteria were diluted to 2,000 CFU and incubated with the indicated concentration of pooled normal human serum (Complement Technology, Inc.). At 0 min and after 30 min of incubation at 37°C, the reaction mixtures were plated for CFU determination. Cells treated with heat-inactivated NHS (NHS^Δi^) for 30 min at 56°C were used as a control. Survival was calculated as the number of colonies recovered at 30 min in active serum-treated samples relative to the number of colonies in samples treated with NHS^Δi^. Experiments were performed in triplicate.

### Protein structure alignment

The Protein Data Bank alignment tool was used to align the structures for Omp26 and Skp. The Omp26 model used was from the *H. influenzae* strain RdKW20 entry AF_AFQ57483F1. The Skp model used was from the *E. coli* strain K12 entry 1SG2. The TM-align alignment method was used to compare the structures.

### Disk diffusion assays

*H. influenzae* strains were grown overnight on CDM or CDM + sub-MIC CLR plates and swabbed into HBSS++. The OD_600_ was adjusted to 1, and the strains were swabbed onto the same medium on which they were grown overnight. Three disks (three technical replicates) with 0.5 mg/mL polymyxin B were applied to each plate. The strains were grown overnight, and two measurements were taken for each zone of inhibition, with three replicates of each strain.

## Data Availability

Sequencing files are available at the National Center for Biotechnology Information Sequence Read Archive (NCBI SRA) under accession number PRJNA514307.

## SUPPLEMENTAL MATERIAL

Supplemental material iai.00369-26-s0001.pdf Legends for Supplemental tables; primer sequences; Fig. S1 and S2, Table S7.

Table S1 iai.00369-26-s0002.xlsx Input gene essentiality analysis for the *H. influenzae* Rd genome.

Table S2 iai.00369-26-s0003.xlsx Resampling fitness data in multiple conditions for *H. influenzae* Rd genome.

Table S3 iai.00369-26-s0004.xlsx Pan-genomic representation of 33 genes involved in clarithromycin resistance.

Table S4 iai.00369-26-s0005.xlsx The list of the 112 genomes used in the pan-genome analysis.

Table S5 iai.00369-26-s0006.xlsx Data from the five Roary pan-genome runs with 112 genomes and a summary of the runs.

Table S6 iai.00369-26-s0007.xlsx Same as Table S5 except Roary pan-genome runs are from 110 genomes.

ASM does not own the copyrights to Supplemental Material that may be linked to, or accessed through, an article. The authors have granted ASM a non-exclusive, world-wide license to publish the Supplemental Material files. Please contact the corresponding author directly for reuse.

## References

[1] Howard AJ, Dunkin KT, Millar GW. 1988. Nasopharyngeal carriage and antibiotic resistance of *Haemophilus influenzae* in healthy children. Epidemiol Infect 100:193–203. doi:10.1017/s0950268800067327 PMC2249230 3258568

[2] Atto B, Kunde D, Gell DA, Tristram S. 2021. Oropharyngeal carriage of *hpl*-Containing *Haemophilus haemolyticus* predicts lower prevalence and density of nthi colonisation in healthy adults. Pathogens 10:577. doi:10.3390/pathogens10050577 PMC8151607 34068621

[3] Finkelstein JA, Davis RL, Dowell SF, Metlay JP, Soumerai SB, Rifas-Shiman SL, Higham M, Miller Z, Miroshnik I, Pedan A, Platt R. 2001. Reducing antibiotic use in children: a randomized trial in 12 practices. Pediatrics 108:1–7. doi:10.1542/peds.108.1.1 11433046

[4] Rabe KF, Watz H. 2017. Chronic obstructive pulmonary disease. The Lancet 389:1931–1940. doi:10.1016/S0140-6736(17)31222-9 28513453

[5] Association AL. 2025. COPD Trends Brief - Mortality. Available from: https://www.lung.org/research/trends-in-lung-disease/copd-trends-brief/copd-mortality

[6] Guarascio AJ, Ray SM, Finch CK, Self TH. 2013. The clinical and economic burden of chronic obstructive pulmonary disease in the USA. Clinicoecon Outcomes Res 5:235–245. doi:10.2147/CEOR.S34321 PMC3694800 23818799

[7] Ahearn CP, Gallo MC, Murphy TF. 2017. Insights on persistent airway infection by non-typeable *Haemophilus influenzae* in chronic obstructive pulmonary disease. Pathog Dis 75:ftx042. doi:10.1093/femspd/ftx042 PMC5437125 28449098

[8] Eldika N, Sethi S. 2006. Role of nontypeable *Haemophilus influenzae* in exacerbations and progression of chronic obstructive pulmonary disease. Curr Opin Pulm Med 12:118–124. doi:10.1097/01.mcp.0000208451.50231.8f 16456381

[9] Siddiqi A, Sethi S. 2008. Optimizing antibiotic selection in treating COPD exacerbations. Int J Chron Obstruct Pulmon Dis 3:31–44. doi:10.2147/copd.s1089 PMC2528209 18488427

[10] Sethi S, Murphy TF. 2008. Infection in the pathogenesis and course of chronic obstructive pulmonary disease. N Engl J Med 359:2355–2365. doi:10.1056/NEJMra0800353 19038881

[11] Short B, Carson S, Devlin A-C, Reihill JA, Crilly A, MacKay W, Ramage G, Williams C, Lundy FT, McGarvey LP, Thornbury KD, Martin SL. 2021. Non-typeable *Haemophilus influenzae* chronic colonization in chronic obstructive pulmonary disease (COPD). Crit Rev Microbiol 47:192–205. doi:10.1080/1040841X.2020.1863330 33455514

[12] Murphy TF, Faden H, Bakaletz LO, Kyd JM, Forsgren A, Campos J, Virji M, Pelton SI. 2009. Nontypeable *Haemophilus influenzae* as a pathogen in children. Pediatr Infect Dis J 28:43–48. doi:10.1097/INF.0b013e318184dba2 19057458

[13] Sethi S, Anzueto A, Miravitlles M, Arvis P, Alder J, Haverstock D, Trajanovic M, Wilson R. 2016. Determinants of bacteriological outcomes in exacerbations of chronic obstructive pulmonary disease. Infection 44:65–76. doi:10.1007/s15010-015-0833-3 PMC4735236 26370552

[14] Van Eldere J, Slack MPE, Ladhani S, Cripps AW. 2014. Non-typeable *Haemophilus influenzae*, an under-recognised pathogen. Lancet Infect Dis 14:1281–1292. doi:10.1016/S1473-3099(14)70734-0 25012226

[15] Block SL, Hedrick J, Tyler R, Smith A, Findlay R, Keegan E, Stroman DW. 2000. Increasing bacterial resistance in pediatric acute conjunctivitis (1997–1998). Antimicrob Agents Chemother 44:1650–1654. doi:10.1128/AAC.44.6.1650-1654.2000 PMC89927 10817723

[16] Medeiros Antone A, O’Brien Thomas F. 1975. Ampicillin-resistant *Haemophilus influenzae* type b possessing a TEM-type β-lactamase but little permeability barrier to ampicillin. The Lancet 305:716–719. doi:10.1016/S0140-6736(75)91630-X 47483

[17] Rubin LG, Yolken RH, Medeiros AA, Richard Moxon E. 1981. Ampicillin treatment failure of apparently β-lactamase-negative *Haemophilus influenzae* type b meningitis due to novel β-lactamase. The Lancet 318:1008–1010. doi:10.1016/S0140-6736(81)91214-9 6118476

[18] Mendelman PM, Chaffin DO, Kalaitzoglou G. 1990. Penicillin-binding proteins and ampicillin resistance in *Haemophilus influenzae*. J Antimicrob Chemother 25:525–534. doi:10.1093/jac/25.4.525 2351623

[19] Malouin F, Parr TR, Bryan LE. 1990. Identification of a group of *Haemophilus influenzae* penicillin-binding proteins that may have complementary physiological roles. Antimicrob Agents Chemother 34:363–365. doi:10.1128/AAC.34.2.363 PMC171589 2327782

[20] Clairoux N, Picard M, Brochu A, Rousseau N, Gourde P, Beauchamp D, Parr TR, Bergeron MG, Malouin F. 1992. Molecular basis of the non-beta-lactamase-mediated resistance to beta-lactam antibiotics in strains of *Haemophilus influenzae* isolated in Canada. Antimicrob Agents Chemother 36:1504–1513. doi:10.1128/AAC.36.7.1504 PMC191612 1510447

[21] Tristram S, Jacobs MR, Appelbaum PC. 2007. Antimicrobial resistance in *Haemophilus influenzae*. Clin Microbiol Rev 20:368–389. doi:10.1128/CMR.00040-06 PMC1865592 17428889

[22] Wilson R, Jones P, Schaberg T, Arvis P, Duprat‐Lomon I, Sagnier PP. 2006. Antibiotic treatment and factors influencing short and long term outcomes of acute exacerbations of chronic bronchitis. Thorax 61:337–342. doi:10.1136/thx.2005.045930 PMC2104610 16449273

[23] Jacobs MR, Bajaksouzian S, Windau A, Good CE, Lin G, Pankuch GA, Appelbaum PC. 2004. Susceptibility of *Streptococcus pneumoniae*, *Haemophilus influenzae*, and *Moraxella catarrhalis* to 17 oral antimicrobial agents based on pharmacodynamic parameters: 1998-2001 U.S. Surveillance Study. Clin Lab Med 24:503–530. doi:10.1016/j.cll.2004.03.008 15177851

[24] Bae S, Lee J, Lee J, Kim E, Lee S, Yu J, Kang Y. 2010. Antimicrobial resistance in *Haemophilus influenzae* respiratory tract isolates in Korea: results of a nationwide acute respiratory infections surveillance. Antimicrob Agents Chemother 54:65–71. doi:10.1128/AAC.00966-09 PMC2798543 19884366

[25] Li J-P, Hua C-Z, Sun L-Y, Wang H-J, Chen Z-M, Shang S-Q. 2017. Epidemiological features and antibiotic resistance patterns of *Haemophilus influenzae* originating from respiratory tract and vaginal specimens in pediatric patients. J Pediatr Adolesc Gynecol 30:626–631. doi:10.1016/j.jpag.2017.06.002 28629795

[26] Wen S, Mai Y, Chen X, Xiao K, Lin Y, Xu Z, Yang L. 2023. Molecular epidemiology and antibiotic resistance analysis of non-typeable *Haemophilus influenzae* (NTHi) in Guangzhou: a representative city of southern China. Antibiotics (Basel) 12:656. doi:10.3390/antibiotics12040656 PMC10135204 37107018

[27] Tsuji BT, Fisher J, Boadi-Yeboah R, Holden PN, Sethi S, Pettigrew MM, Murphy TF. 2018. Azithromycin pharmacodynamics against persistent *Haemophilus influenzae* in chronic obstructive pulmonary disease. Antimicrob Agents Chemother 62:01995–17. doi:10.1128/AAC.01995-17 PMC5786777 29180527

[28] Champney WS, Miller M. 2002. Inhibition of 50S ribosomal subunit assembly in *Haemophilus influenzae* cells by azithromycin and erythromycin. Curr Microbiol 44:418–424. doi:10.1007/s00284-001-0016-6 12000992

[29] Hrvacić B, Bosnjak B, Bosnar M, Ferencić Z, Glojnarić I, Eraković Haber V. 2009. Clarithromycin suppresses airway hyperresponsiveness and inflammation in mouse models of asthma. Eur J Pharmacol 616:236–243. doi:10.1016/j.ejphar.2009.06.032 19560456

[30] Nakamura S, Yanagihara K, Araki N, Yamada K, Morinaga Y, Izumikawa K, Seki M, Kakeya H, Yamamoto Y, Kamihira S, Kohno S. 2010. Efficacy of clarithromycin against experimentally induced pneumonia caused by clarithromycin-resistant *Haemophilus influenzae* in mice. Antimicrob Agents Chemother 54:757–762. doi:10.1128/AAC.00524-09 PMC2812181 19949056

[31] Ivetić Tkalcević V, Bosnjak B, Hrvacić B, Bosnar M, Marjanović N, Ferencić Z, Situm K, Culić O, Parnham MJ, Eraković V. 2006. Anti-inflammatory activity of azithromycin attenuates the effects of lipopolysaccharide administration in mice. Eur J Pharmacol 539:131–138. doi:10.1016/j.ejphar.2006.03.074 16698012

[32] Matsuoka N, Eguchi K, Kawakami A, Tsuboi M, Kawabe Y, Aoyagi T, Nagataki S. 1996. Inhibitory effect of clarithromycin on costimulatory molecule expression and cytokine production by synovial fibroblast-like cells. Clin Exp Immunol 104:501–508. doi:10.1046/j.1365-2249.1996.46752.x PMC2200457 9099936

[33] Seyama S, Wajima T, Nakaminami H, Noguchi N. 2017. Amino acid substitution in the major multidrug efflux transporter protein acrB contributes to low susceptibility to azithromycin in *Haemophilus influenzae*. Antimicrob Agents Chemother 61:01337–17. doi:10.1128/AAC.01337-17 PMC5655073 28848006

[34] Cadenas-Jiménez I, Saiz-Escobedo L, Carrera-Salinas A, Camprubí-Márquez X, Calvo-Silveria S, Camps-Massa P, Berbel D, Tubau F, Santos S, Domínguez MA, González-Díaz A, Ardanuy C, Martí S. 2024. Molecular characterization of macrolide resistance in *Haemophilus influenzae* and *Haemophilus parainfluenzae* strains (2018-21). J Antimicrob Chemother 79:2194–2203. doi:10.1093/jac/dkae214 38946313

[35] Hansen JL, Ippolito JA, Ban N, Nissen P, Moore PB, Steitz TA. 2002. The structures of four macrolide antibiotics bound to the large ribosomal subunit. Mol Cell 10:117–128. doi:10.1016/s1097-2765(02)00570-1 12150912

[36] Zaman S, Fitzpatrick M, Lindahl L, Zengel J. 2007. Novel mutations in ribosomal proteins L4 and L22 that confer erythromycin resistance in *Escherichia coli*. Mol Microbiol 66:1039–1050. doi:10.1111/j.1365-2958.2007.05975.x PMC2229831 17956547

[37] Wekselman I, Zimmerman E, Davidovich C, Belousoff M, Matzov D, Krupkin M, Rozenberg H, Bashan A, Friedlander G, Kjeldgaard J, Ingmer H, Lindahl L, Zengel JM, Yonath A. 2017. The ribosomal protein uL22 modulates the shape of the protein exit tunnel. Structure 25:1233–1241. doi:10.1016/j.str.2017.06.004 28689968

[38] Schlünzen F, Zarivach R, Harms J, Bashan A, Tocilj A, Albrecht R, Yonath A, Franceschi F. 2001. Structural basis for the interaction of antibiotics with the peptidyl transferase centre in eubacteria. Nature 413:814–821. doi:10.1038/35101544 11677599

[39] Sánchez L, Pan W, Viñas M, Nikaido H. 1997. The acrAB homolog of *Haemophilus influenzae* codes for a functional multidrug efflux pump. J Bacteriol 179:6855–6857. doi:10.1128/jb.179.21.6855-6857.1997 PMC179619 9352940

[40] Gawronski JD, Wong SMS, Giannoukos G, Ward DV, Akerley BJ. 2009. Tracking insertion mutants within libraries by deep sequencing and a genome-wide screen for *Haemophilus* genes required in the lung. Proc Natl Acad Sci USA 106:16422–16427. doi:10.1073/pnas.0906627106 PMC2752563 19805314

[41] Goodman AL, McNulty NP, Zhao Y, Leip D, Mitra RD, Lozupone CA, Knight R, Gordon JI. 2009. Identifying genetic determinants needed to establish a human gut symbiont in its habitat. Cell Host Microbe 6:279–289. doi:10.1016/j.chom.2009.08.003 PMC2895552 19748469

[42] van Opijnen T, Bodi KL, Camilli A. 2009. Tn-seq: high-throughput parallel sequencing for fitness and genetic interaction studies in microorganisms. Nat Methods 6:767–772. doi:10.1038/nmeth.1377 PMC2957483 19767758

[43] Langridge GC, Phan M-D, Turner DJ, Perkins TT, Parts L, Haase J, Charles I, Maskell DJ, Peters SE, Dougan G, Wain J, Parkhill J, Turner AK. 2009. Simultaneous assay of every *Salmonella typhi* gene using one million transposon mutants. Genome Res 19:2308–2316. doi:10.1101/gr.097097.109 PMC2792183 19826075

[44] Herriott RM, Meyer EY, Vogt M, Modan M. 1970. Defined medium for growth of *Haemophilus influenzae*. J Bacteriol 101:513–516. doi:10.1128/jb.101.2.513-516.1970 PMC284935 5308770

[45] Harrison A, Ray WC, Baker BD, Armbruster DW, Bakaletz LO, Munson RS. 2007. The OxyR regulon in nontypeable *Haemophilus influenzae*. J Bacteriol 189:1004–1012. doi:10.1128/JB.01040-06 PMC1797302 17142400

[46] Morton DJ, Seale TW, Madore LL, VanWagoner TM, Whitby PW, Stull TL. 2007. The haem-haemopexin utilization gene cluster (*hxuCBA*) as a virulence factor of *Haemophilus influenzae*. Microbiology 153:215–224. doi:10.1099/mic.0.2006/000190-0 17185550

[47] Liou G-G, Chang H-Y, Lin C-S, Lin-Chao S. 2002. DEAD box RhlB RNA helicase physically associates with exoribonuclease PNPase to degrade double-stranded RNA independent of the degradosome-assembling region of RNase E. J Biol Chem 277:41157–41162. doi:10.1074/jbc.M206618200 12181321

[48] Khemici V, Poljak L, Toesca I, Carpousis AJ. 2005. Evidence *in vivo* that the DEAD-box RNA helicase RhlB facilitates the degradation of ribosome-free mRNA by RNase E. Proc Natl Acad Sci U S A 102:6913–6918. doi:10.1073/pnas.0501129102 PMC1100780 15867149

[49] Zetzsche H, Raschke L, Fürtig B. 2023. Allosteric activation of RhlB by RNase E induces partial duplex opening in substrate RNA. Front Mol Biosci 10:1139919. doi:10.3389/fmolb.2023.1139919 PMC10500059 37719267

[50] Bernstein JA, Lin P-H, Cohen SN, Lin-Chao S. 2004. Global analysis of *Escherichia coli* RNA degradosome function using DNA microarrays. Proc Natl Acad Sci USA 101:2758–2763. doi:10.1073/pnas.0308747101 PMC365694 14981237

[51] Hood DW, Cox AD, Wakarchuk WW, Schur M, Schweda EKH, Walsh SL, Deadman ME, Martin A, Moxon ER, Richards JC. 2001. Genetic basis for expression of the major globotetraose-containing *Lipopolysaccharide* from *H. influenzae* strain Rd (RM118). Glycobiology 11:957–967. doi:10.1093/glycob/11.11.957 11744630

[52] Langereis JD, Stol K, Schweda EK, Twelkmeyer B, Bootsma HJ, Vries SPW, Burghout P, Diavatopoulos DA, Hermans PWM. 2012. Modified lipooligosaccharide structure protects nontypeable *Haemophilus influenzae* from IgM-mediated complement killing in experimental otitis media. mBio. doi:10.1128/mBio.00079-12 PMC3398534 22761391

[53] Kyd JM, Cripps AW. 1998. Potential of a novel protein, OMP26, from nontypeable *Haemophilus influenzae* to enhance pulmonary clearance in a rat model. Infect Immun 66:2272–2278. doi:10.1128/IAI.66.5.2272-2278.1998 PMC108191 9573117

[54] Vogel AR, Szelestey BR, Raffel FK, Sharpe SW, Gearinger RL, Justice SS, Mason KM. 2012. SapF-mediated heme-iron utilization enhances persistence and coordinates biofilm architecture of *Haemophilus*. Front Cell Infect Microbiol 2:42. doi:10.3389/fcimb.2012.00042 PMC3417626 22919633

[55] Nakamura S, Shchepetov M, Dalia AB, Clark SE, Murphy TF, Sethi S, Gilsdorf JR, Smith AL, Weiser JN. 2011. Molecular basis of increased serum resistance among pulmonary isolates of non-typeable *Haemophilus influenzae*. PLoS Pathog 7:e1001247. doi:10.1371/journal.ppat.1001247 PMC3017122 21253576

[56] Hogg JS, Hu FZ, Janto B, Boissy R, Hayes J, Keefe R, Post JC, Ehrlich GD. 2007. Characterization and modeling of the *Haemophilus influenzae* core and supragenomes based on the complete genomic sequences of Rd and 12 clinical nontypeable strains. Genome Biol 8:R103. doi:10.1186/gb-2007-8-6-r103 PMC2394751 17550610

[57] Pinto M, González-Díaz A, Machado MP, Duarte S, Vieira L, Carriço JA, Marti S, Bajanca-Lavado MP, Gomes JP. 2019. Insights into the population structure and pan-genome of *Haemophilus influenzae*. Infect Genet Evol 67:126–135. doi:10.1016/j.meegid.2018.10.025 30391557

[58] De Chiara M, Hood D, Muzzi A, Pickard DJ, Perkins T, Pizza M, Dougan G, Rappuoli R, Moxon ER, Soriani M, Donati C. 2014. Genome sequencing of disease and carriage isolates of nontypeable *Haemophilus influenzae* identifies discrete population structure. Proc Natl Acad Sci U S A 111:5439–5444. doi:10.1073/pnas.1403353111 PMC3986186 24706866

[59] Page AJ, Cummins CA, Hunt M, Wong VK, Reuter S, Holden MTG, Fookes M, Falush D, Keane JA, Parkhill J. 2015. Roary: rapid large-scale prokaryote pan genome analysis. Bioinformatics 31:3691–3693. doi:10.1093/bioinformatics/btv421 PMC4817141 26198102

[60] Lambert RJW, Pearson J. 2000. Susceptibility testing: accurate and reproducible minimum inhibitory concentration (MIC) and non-inhibitory concentration (NIC) values. J Appl Microbiol 88:784–790. doi:10.1046/j.1365-2672.2000.01017.x 10792538

[61] Seyama S, Wajima T, Nakaminami H, Noguchi N. 2016. Clarithromycin resistance mechanisms of epidemic β-lactamase-nonproducing ampicillin-resistant *Haemophilus influenzae* strains in Japan. Antimicrob Agents Chemother 60:3207–3210. doi:10.1128/AAC.00163-16 PMC4862528 26953210

[62] Trepod CM, Mott JE. 2004. Identification of the *Haemophilus influenzae tolC* gene by susceptibility profiles of insertionally inactivated efflux pump mutants. Antimicrob Agents Chemother 48:1416–1418. doi:10.1128/AAC.48.4.1416-1418.2004 PMC375248 15047557

[63] CLSI. Breakpoint implementation toolkit. 2024 edition. CLSI Guideline.

[64] Risberg A, Alvelius G, Schweda EKH. 1999. Structural analysis of the lipopolysaccharide oligosaccharide epitopes expressed by *Haemophilus influenzae* strain RM.118-26. Eur J Biochem 265:1067–1074. doi:10.1046/j.1432-1327.1999.00832.x 10518803

[65] Hood DW, Makepeace K, Deadman ME, Rest RF, Thibault P, Martin A, Richards JC, Moxon ER. 1999. Sialic acid in the lipopolysaccharide of *Haemophilus influenzae*: strain distribution, influence on serum resistance and structural characterization. Mol Microbiol 33:679–692. doi:10.1046/j.1365-2958.1999.01509.x 10447878

[66] Wong Sandy M. S., St. Michael F, Cox A, Ram S, Akerley BJ. 2011. ArcA-regulated glycosyltransferase Lic2B promotes complement evasion and pathogenesis of nontypeable *Haemophilus influenzae*. Infect Immun 79:1971–1983. doi:10.1128/IAI.01269-10 PMC3088160 21357723

[67] Peric M, Bozdogan B, Jacobs MR, Appelbaum PC. 2003. Effects of an efflux mechanism and ribosomal mutations on macrolide susceptibility of *Haemophilus influenzae* clinical isolates. Antimicrob Agents Chemother 47:1017–1022. doi:10.1128/AAC.47.3.1017-1022.2003 PMC149331 12604536

[68] Bogdanovich T, Bozdogan B, Appelbaum PC. 2006. Effect of efflux on telithromycin and macrolide susceptibility in *Haemophilus influenzae*. Antimicrob Agents Chemother 50:893–898. doi:10.1128/AAC.50.3.893-898.2006 PMC1426444 16495248

[69] Jánosity A, Klančnik A, Kiskó G, Možina SS, Baranyi J. 2021. Determining optimum carvacrol treatment as a cardinal value of a secondary model. Int J Food Microbiol 354:109311. doi:10.1016/j.ijfoodmicro.2021.109311 34225033

[70] Zhang Y, Skolnick J. 2005. TM-align: a protein structure alignment algorithm based on the TM-score. Nucleic Acids Res 33:2302–2309. doi:10.1093/nar/gki524 PMC1084323 15849316

[71] Bittrich S, Segura J, Duarte JM, Burley SK, Rose Y. 2024. RCSB protein Data Bank: exploring protein 3D similarities via comprehensive structural alignments. Bioinformatics 40:btae370. doi:10.1093/bioinformatics/btae370 PMC11212067 38870521

[72] Korndörfer IP, Dommel MK, Skerra A. 2004. Structure of the periplasmic chaperone Skp suggests functional similarity with cytosolic chaperones despite differing architecture. Nat Struct Mol Biol 11:1015–1020. doi:10.1038/nsmb828 15361861

[73] Varadi M, Anyango S, Deshpande M, Nair S, Natassia C, Yordanova G, Yuan D, Stroe O, Wood G, Laydon A, et al.. 2022. Alphafold protein structure database: massively expanding the structural coverage of protein-sequence space with high-accuracy models. Nucleic Acids Res 50:D439–D444. doi:10.1093/nar/gkab1061 PMC8728224 34791371

[74] Varadi M, Bertoni D, Magana P, Paramval U, Pidruchna I, Radhakrishnan M, Tsenkov M, Nair S, Mirdita M, Yeo J, Kovalevskiy O, Tunyasuvunakool K, Laydon A, Žídek A, Tomlinson H, Hariharan D, Abrahamson J, Green T, Jumper J, Birney E, Steinegger M, Hassabis D, Velankar S. 2024. Providing structure coverage for over 214 million protein sequences. Nucleic Acids Res 52:D368–D375. doi:10.1093/nar/gkad1011 PMC10767828 37933859

[75] Murray JL, Kwon T, Marcotte EM, Whiteley M. 2015. Intrinsic antimicrobial resistance determinants in the superbug *Pseudomonas aeruginosa*. mBio 6. doi:10.1128/mBio.01603-15 PMC4626858 26507235

[76] Saxena D, Maitra R, Bormon R, Czekanska M, Meiers J, Titz A, Verma S, Chopra S. 2023. Tackling the outer membrane: facilitating compound entry into gram-negative bacterial pathogens. NPJ Antimicrob Resist 1:17. doi:10.1038/s44259-023-00016-1 PMC11721184 39843585

[77] Zhang Q-Y, Yan Z-B, Meng Y-M, Hong X-Y, Shao G, Ma J-J, Cheng X-R, Liu J, Kang J, Fu C-Y. 2021. Antimicrobial peptides: mechanism of action, activity and clinical potential. Mil Med Res 8:48. doi:10.1186/s40779-021-00343-2 PMC8425997 34496967

[78] Gallagher LA, Lee SA, Manoil C. 2017. Importance of core genome functions for an extreme antibiotic resistance trait. mBio 8:e01655-17. doi:10.1128/mBio.01655-17 PMC5727411 29233894

[79] Zähner D, Zhou X, Chancey ST, Pohl J, Shafer WM, Stephens DS. 2010. Human antimicrobial peptide LL-37 induces MefE/Mel-mediated macrolide resistance in *Streptococcus pneumoniae*. Antimicrob Agents Chemother 54:3516–3519. doi:10.1128/AAC.01756-09 PMC2916318 20498319

[80] Fouts DE, Clarke TH, Severin GB, Brown AN, Ottosen EN, Holmes CL, Moricz BS, Mason S, Sinha R, Anderson MT, DiRita V, Bachman MA, Mobley HLT. 2025. Integrating genomic and Tn-Seq data to identify common *in vivo* fitness mechanisms across multiple bacterial species. mBio 16:e0198825. doi:10.1128/mbio.01988-25 PMC12607656 40981419

[81] Xu W, DeJesus MA, Rücker N, Engelhart CA, Wright MG, Healy C, Lin K, Wang R, Park SW, Ioerger TR, Schnappinger D, Ehrt S. 2017. Chemical genetic interaction profiling reveals determinants of intrinsic antibiotic resistance in *Mycobacterium tuberculosis*. Antimicrob Agents Chemother 61:01334–17. doi:10.1128/AAC.01334-17 PMC5700314 28893793

[82] Choi N, Choi E, Cho Y-J, Kim MJ, Choi HW, Lee E-J. 2024. A shared mechanism of multidrug resistance in laboratory-evolved uropathogenic *Escherichia coli*. Virulence 15:2367648. doi:10.1080/21505594.2024.2367648 PMC11195483 38899601

[83] Olliver A, Vallé M, Chaslus-Dancla E, Cloeckaert A. 2004. Role of an *acrR* mutation in multidrug resistance of *in vitro*-selected fluoroquinolone-resistant mutants of *Salmonella enterica* serovar typhimurium. FEMS Microbiol Lett 238:267–272. doi:10.1016/j.femsle.2004.07.046 15336432

[84] Delcour AH. 2009. Outer membrane permeability and antibiotic resistance. Biochimica et Biophysica Acta (BBA) - Proteins and Proteomics 1794:808–816. doi:10.1016/j.bbapap.2008.11.005 PMC2696358 19100346

[85] Loeb MR, Smith DH. 1980. Outer membrane protein composition in disease isolates of *Haemophilus influenzae*: pathogenic and epidemiological implications. Infect Immun 30:709–717. doi:10.1128/iai.30.3.709-717.1980 PMC551373 6971807

[86] van Alphen L, Riemens T, Poolman J, Zanen HC. 1983. Characteristics of major outer membrane proteins of *Haemophilus influenzae*. J Bacteriol 155:878–885. doi:10.1128/jb.155.2.878-885.1983 PMC217763 6603458

[87] Kannan K, Vázquez-Laslop N, Mankin AS. 2012. Selective protein synthesis by ribosomes with a drug-obstructed exit tunnel. Cell 151:508–520. doi:10.1016/j.cell.2012.09.018 23101624

[88] Naganathan A, Wood MP, Moore SD. 2015. The large ribosomal subunit protein L9 enables the growth of EF-P deficient cells and enhances small subunit maturation. PLoS One 10:e0120060. doi:10.1371/journal.pone.0120060 PMC4399890 25879934

[89] Gupta P, Kannan K, Mankin AS, Vázquez-Laslop N. 2013. Regulation of gene expression by macrolide-induced ribosomal frameshifting. Mol Cell 52:629–642. doi:10.1016/j.molcel.2013.10.013 PMC3874817 24239289

[90] Kohanski MA, Dwyer DJ, Hayete B, Lawrence CA, Collins JJ. 2007. A common mechanism of cellular death induced by bactericidal antibiotics. Cell 130:797–810. doi:10.1016/j.cell.2007.06.049 17803904

[91] Dwyer DJ, Belenky PA, Yang JH, MacDonald IC, Martell JD, Takahashi N, Chan CTY, Lobritz MA, Braff D, Schwarz EG, Ye JD, Pati M, Vercruysse M, Ralifo PS, Allison KR, Khalil AS, Ting AY, Walker GC, Collins JJ. 2014. Antibiotics induce redox-related physiological alterations as part of their lethality. Proc Natl Acad Sci USA 111. doi:10.1073/pnas.1401876111 PMC4034191 24803433

[92] Keren I, Wu Y, Inocencio J, Mulcahy LR, Lewis K. 2013. Killing by bactericidal antibiotics does not depend on reactive oxygen species. Science 339:1213–1216. doi:10.1126/science.1232688 23471410

[93] Liu Y, Imlay JA. 2013. Cell death from antibiotics without the involvement of reactive oxygen species. Science 339:1210–1213. doi:10.1126/science.1232751 PMC3731989 23471409

[94] Smith HK, Nelson KL, Calaunan ES, Smith AL, Nguyen V. 2013. “Affect of anaerobiosis on the antibiotic susceptibility of *H. influenzae*”. BMC Res Notes 6:241. doi:10.1186/1756-0500-6-241 PMC3723416 23803418

[95] Kim G, Weiss SJ, Levine RL. 2014. Methionine oxidation and reduction in proteins. Biochim Biophys Acta 1840:901–905. doi:10.1016/j.bbagen.2013.04.038 PMC3766491 23648414

[96] Gruber CC, Walker GC. 2018. Incomplete base excision repair contributes to cell death from antibiotics and other stresses. DNA Repair (Amst) 71:108–117. doi:10.1016/j.dnarep.2018.08.014 PMC6442677 30181041

[97] Wong SMS, Gawronski JD, Lapointe D, Akerley BJ. 2011. High-throughput insertion tracking by deep sequencing for the analysis of bacterial pathogens, p 209–222. In Kwon YM, Ricke SC (ed), High-Throughput Next Generation Sequencing: Methods and Applications. Humana Press, Totowa, NJ. 10.1007/978-1-61779-089-8_15 21431773

[98] Barcak GJ, Chandler MS, Redfield RJ, Tomb J-F. 1991. Genetic systems in *Haemophilus influenzae*, p 321–342. *In* In Methods in Enzymology. Academic Press. 10.1016/0076-6879(91)04016-h 1943781

[99] DeJesus MA, Ambadipudi C, Baker R, Sassetti C, Ioerger TR. 2015. TRANSIT--a software tool for himar1 tnseq analysis. PLoS Comput Biol 11:e1004401. doi:10.1371/journal.pcbi.1004401 PMC4598096 26447887

[100] Sambrook J, Fritsch E, Maniatis T. 1989. Molecular cloning: a laboratory manual. 2nd ed. Cold Spring Harbor Laboratory Press, Cold Spring Harbor, NY.

[101] Fleischmann RD, Adams MD, White O, Clayton RA, Kirkness EF, Kerlavage AR, Bult CJ, Tomb J-F, Dougherty BA, Merrick JM. 1995. Whole-genome random sequencing and assembly of *Haemophilus influenzae* Rd. Science 269:496–512. doi:10.1126/science.7542800 7542800

[102] Wong SM, Akerley BJ. 2003. Inducible expression system and marker-linked mutagenesis approach for functional genomics of *Haemophilus influenzae*. Gene 316:177–186. doi:10.1016/S0378-1119(03)00762-5 14563564

[103] Wong SMS, Alugupalli KR, Ram S, Akerley BJ. 2007. The ArcA regulon and oxidative stress resistance in *Haemophilus influenzae*. Mol Microbiol 64:1375–1390. doi:10.1111/j.1365-2958.2007.05747.x PMC1974803 17542927

[104] Paixão L, Rodrigues L, Couto I, Martins M, Fernandes P, de Carvalho CC, Monteiro GA, Sansonetty F, Amaral L, Viveiros M. 2009. Fluorometric determination of ethidium bromide efflux kinetics in *Escherichia coli*. J Biol Eng 3. doi:10.1186/1754-1611-3-18 PMC2774284 19835592

