# Supplementary material for: Dual roles of genes required for intrinsic resistance to clarithromycin in evasion of killing by serum complement in Haemophilus influenzae
Source: Infect Immun. 2026 Aug 6;94(9):e00369-26. doi: 10.1128/iai.00369-26 (PMC13552136; doi:10.1128/iai.00369-26)
Supplement: Supplementary file 1 [file iai.00369-26-s0001.pdf]

TEXT S1

<u>Primer Sequences</u>

Name	Sequence
adap1	5’-AGTTCTCCAGGTCTTGCGTTGCTCTTCCGATC*T-3’
adap3	5’-p-GATCGGAAGAGCACACGTCTGAACTCCAGTCAC-3’
marout-nest 2	5’-AGACCGGGGACTTATCAG-3’
oljJD	5’-GTGACTGGAGTTCAGACGT*G-3’
MarPCR2-5N	5’-AATGATACGGCGACCACCGAGATCTACACTCTTTCCCTACACGACGCTCTTCCGATCTNNNNNCCGGGGACTTATCAGCCAAC*C -3’
BC__	5’-CAAGCAGAAGACGGCATACGAGATxxxxxxGTGACTGGAGTTCAGACGTGTG -3’
5'-HI0916-omp26	5'-CTATCAAACTTATACAGCGGGTGG-3'
3'-HI0916-omp26-gen	5'-AACATCGTTGCTGCTGCGTAACATTTGATAAATATCCTTAATTAAATGATGGGTTTAAT-3'
5'-HI0916-omp26-gen	5'-ATCGACCCAAGTACCGCCACCTAATAGGTTCTCATAATGCAAAAATCCTATTCTTT-3'
3'-HI0916-omp26	5'-CCATCACTACCGATAACTGTTCC-3'
5_HI0461_1	5'-TCATCGCCATAAGTGGTTTCG-3'
3_HI0461_1g	5'-AACATCGTTGCTGCTGCGTAACATAACTCAATATTTTGTGAAATTTAATAAAATTTTTC-3'
5_HI0461_2g	5'-ATCGACCCAAGTACCGCCACCTAACGAAGAAAGTGCGGTAAAAAATGAGAG-3'
3_HI0461_2	5'-GCAAGTCGTTTTCTTCAGTCAG-3'
5'-HI0916-out	5'-AGCATCTGCAGGATATGCAAATGG-3'
3'-HI0916-out	5'-CGCACCAATCTCAACGTTATTACC-3'
5'-HI0461-out	5'-AGGGCATTGAGAATATCTGTAGGC-3'

3'-HI0461-out	5'-CCAAATTTACGCGAAGAATTAGGGG-3'
5Gent2	5'-ATGTTACGCAGCAGCAACGATGTT-3'
3Gent2	5'-TTAGGTGGCGGTACTTGGGTCGAT-3'
HI0524-int5’	5’-CAAAAAGTTTTTGCTTATGCTAAAG-3’
HI0521-int3’	5’-TAAATCGCGTAGCCTTCACATAAATC-3’
HI0524-5’-orf	5'-ATGGCTAAATTATTAGATATTG-3’
HI0521-3’-orf	5'-TTTTTCAGCGATCACATC-3’
5-HI0916-Kan	5’-CATTTGATGCTCGATGAGTTTTTCTAATAATAGGTTCTCATAATGCAAAAATCCT-3’
3-HI0916-Kan	5’-TTCCCGTTGAATATGGCTCATCATTTGATAAATATCCTTAATTAAATGATGG-3’
5Kan1.1	5’-ATGAGCCATATTCAACGGGAA-3’
3Kan1.1	5’-TTAGAAAAACTCATCGAGCATCAAATG-3’
acrR_d141nt_F	5'-ACCTTTATTTAAAAACAAAGATGAGTTGC-3’
acrR_R	5'-TGTTGTGTTAATAACTGGCGTCC-3’
acrR_d141nt_R	5'-CTTTGTTTTTAAATAAAGGTAAATCGTTCC-3’
acrR_F	5'-GGTTTACTGACTGGCGATGTAG-3’
3-HI0523-SapI	5'-TTGCTCTTCGTTAGCGGCCGCTTCTTAAATATTATTGCTCGTTTGCCACA-3’
5-HI0523-SapI	5'-TTGCTCTTCTATGAGTGCGGTTTTTTTATTTTAGCGGA-3’
3-HI0916-SapI	5'-TTGCTCTTCGTTAGCGGCCGCTATTATTTTTTCTCTTGTGCTTTTTCAG-3’
5-HI0916-SapI	5'-TTGCTCTTCAATGAAAAACATCGCAAAAGTAAC-3’

[a] * = phosphorothioate linkage, [b] p= 5’ phosphorylation, [c] xxxxxx = 6bp Illumina TruSeq barcode sequence

Table S1

Input gene essentiality analysis for the *H. influenzae* Rd genome using the Hidden Markov Model (HMM) analysis in which genes were called as essential (E), nonessential (NE), growth defective (GD), or growth advantaged (GA). Columns in the table are as follows: Locus Tag (HI number), Gene (gene name), Description (protein information), No. TA sites (Number of TA sites), No. sites ES (Number of essential sites), No. sites GD (Number of growth defective sites), No. sites NE (Number of nonessential sites), No. sites GA (Number of growth advantaged sites), Ave. insertion rate within gene (Mean insertion rate within the gene), Mean read count within gene, and Call (classified as either E, NE, GD, or GA).

Table S2

Resampling fitness data in High CLR, Low CLR, CDM + Solvent, CDM, and the murine lung infection for the *H. influenzae* Rd genome. Columns in the table are Locus Tag (HI number); Gene (gene name); Description (protein information); the log2FC (log2FoldChange), p-value, and Adj. p-value (adjusted p-value calculated using the Benjamini-Hochberg procedure) for High CLR, Low CLR, CDM + Solvent, CDM, and the murine lung infection; KEGG (Kyoto Encyclopedia of Genes and Genomes pathways); and Individual Essentiality calls—essential (E), non-essential (NE), growth defective (GD), or growth advantaged (GA)—for each gene based on the input data from Table S1. P-values and adjusted p-values that are 0 represent values less than 0.000001 that were truncated at 5 decimal places.

Table S3

Pan-genomic representation of 33 genes involved in clarithromycin resistance identified. Columns in the table are Locus Tag (HI number); Gene (gene name); Description (protein information); the log2FC

(log2FoldChange), p-value, and Adj. p-value (adjusted p-value calculated using the Benjamini-Hochberg procedure) for High CLR, Low CLR, CDM + Solvent, CDM, and the murine lung infection; KEGG (Kyoto Encyclopedia of Genes and Genomes pathways); and Individual Essentiality calls—essential (E), non-essential (NE), growth defective (GD), or growth advantaged (GA)—for each gene based on the input data from Table S1; categories for each gene (transporters/efflux, transcription and translation, cell wall and outer membrane, oxidative stress genes, uncategorized); Roary run 1-5: pan-genome outputs of number of genomes containing the gene; Core Gene? pan-genome call: core 99% <= genomes <= 100%, outer core 95% <= genomes < 99%, or shell 15% <= genomes < 95%); and the Pan-genome Mode: the mode of the Roary runs.

Table S4

The list of the 112 genomes that were used in the pan-genome analysis with the assembly name and genome name.

Table S5

Data from the five Roary pan-genome runs with 112 genomes and a summary of the runs. The first five sheets contain the data from the five runs. The columns in the first five sheets are Order (order of the data output from Roary), Gene (unique gene name from Roary), Non.unique.Gene.name (gene name for genes that received a “group name” in the Gene column), Annotation (protein information from Roary), No. isolates (number of genomes containing the gene), columns F-DM are labeled with genome names and each column contains the Roary gene ID. See https://sangerpathogens.github.io/Roary/ for more information. The columns in the summary are Locus Tag (HI number); Gene (gene name); Description

(protein information), Roary run 1-5 (pan-genome outputs of number of genomes containing the gene), and Runs Agree? (whether the different runs called the same number of genomes containing the gene).

<u>Table S6</u>

Same description as for Table S5 except Roary pan-genome runs are from 110 genomes excluding genomes 5P54H1 and 17 (columns F-DK).

<u>Table S7. MIC Validation.</u>

**Strain**	**MIC (µg/mL)**
RdAW	6.3
Rd**Δ**0461	3.1
Rd**Δ**orfH	3.1
Rd**Δ**omp26	6.3
Hi375	12.5
375**Δ**0461	6.3
375**Δ**orfH	3.1
375**Δ**omp26	3.1-6.3

Each strain was grown in CDM in presence of 2-fold dilutions of CLR to determine the minimum inhibitory concentration. n=3-6. Standard error of the mean was 0 for all strains except 375Δomp26, which was 0.659.

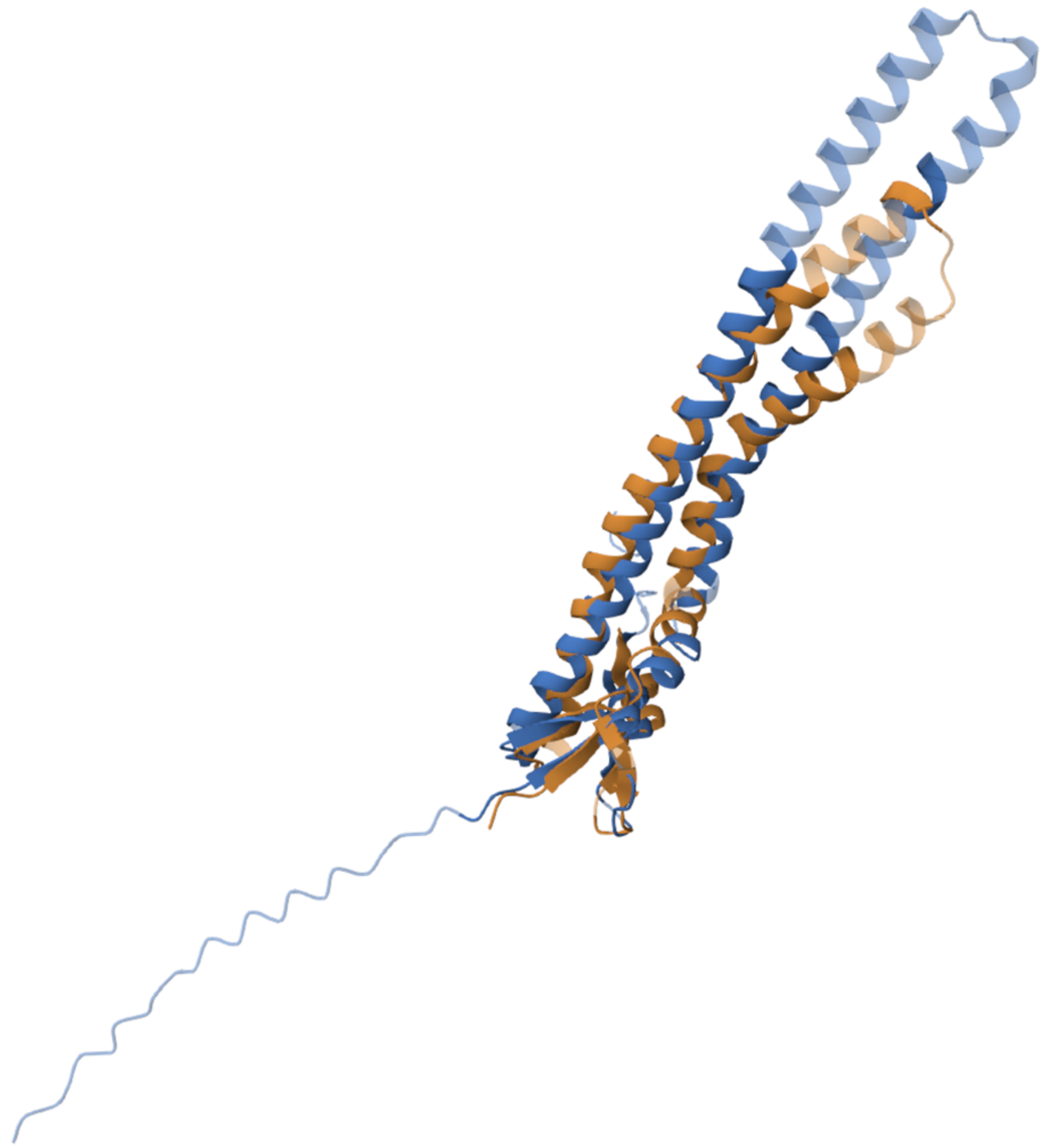

Figure S1. Omp26 and Skp structural alignment using the RCSB Protein Data Bank. The Omp26 model (blue) is a computationally derived structure from AlphaFold and is in its unprocessed form. The Skp model (brown) was experimentally-derived using crystallography (1).

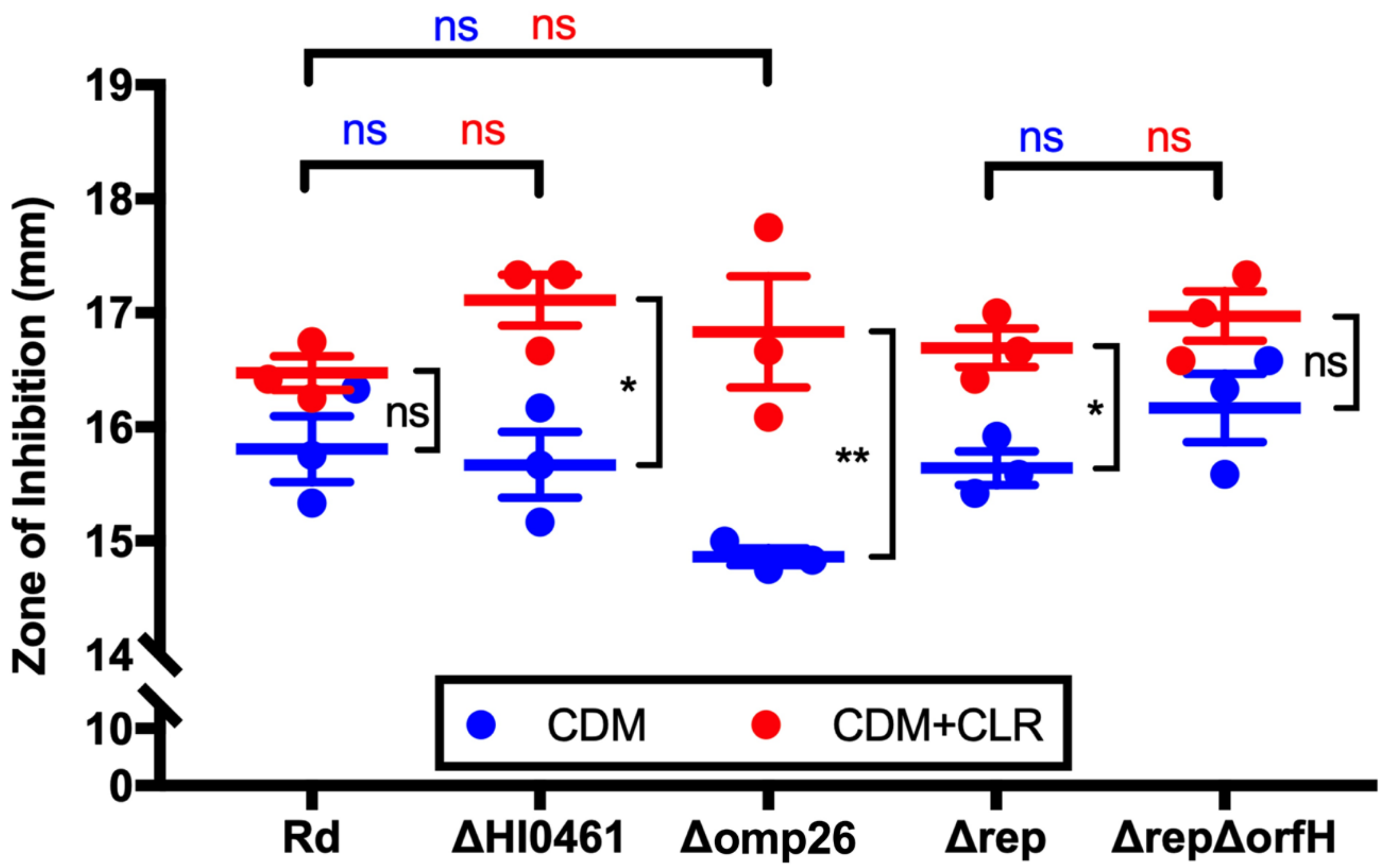

Figure S2. Pretreatment with clarithromycin increased sensitivity of RdAW deletion mutants to polymyxin B. *H. influenzae* parental strains Rd and RdΔrep and their indicated isogenic mutants were grown on either CDM or CDM + sub-MIC CLR (CDM+CLR) plates with disks containing 0.5 mg/mL polymyxin B. Zones of inhibitions were measured after overnight incubation. Differences were evaluated via ANOVA with Šidák's multiple comparisons test (*, P<0.05; **, P<0.005; ns, not statistically significant). Δrep is a LicA locked phase derivative in Rd (2); ΔrepΔorfH contains an *orfH* deletion in parent strain Δrep created by introducing an *orfH* knockout PCR product as described (3 and Materials and Methods).

Roary *acrR* (HI0893) discrepancy

Roary called *acrR* as present in only 74 or 91 out of 112 genomes, depending on the run. 2 of the 112 genomes have two copies of *acrR* (HI0893). The sequences for these genes both contain early stop codons in *acrR*. And these sequences are annotated as having an in-frame methionine present that could allow reinitiation of translation, potentially leading to truncated versions of *acrR*. Therefore, we removed these 2 genomes (5P54H1 and 17) with 2 copies of *acrR* and re-ran Roary. These runs called *acrR* as present in 107 out of 110 genomes (Table S7). So, we conclude that *acrR* falls in the outer core. This type of discrepancy occurred for 92 other genes (Table S6), where the number of genomes containing the gene is different depending on the run. These discrepancies between Roary runs can be used an indicator that the genomes may have errors. The *acrR* discrepancy is the only occurrence of a discrepancy in more than 1 genome for the 33 genes involved in CLR resistance we identified, however other occurrences would have to be dealt with on an individual basis.

Supplemental References

1. Korndörfer IP, Dommel MK, Skerra A. 2004. Structure of the periplasmic chaperone Skp suggests functional similarity with cytosolic chaperones despite differing architecture. Nat Struct Mol Biol 11:1015–1020.
2. Wong SM, Akerley BJ. 2005. Environmental and genetic regulation of the phosphorylcholine epitope of *Haemophilus influenzae* lipooligosaccharide. Molecular Microbiology 55:724–738.

3. Gawronski JD, Wong SMS, Giannoukos G, Ward DV, Akerley BJ. 2009. Tracking insertion mutants within libraries by deep sequencing and a genome-wide screen for *Haemophilus* genes required in the lung. Proc Natl Acad Sci U S A 106:16422–16427.
